# Smad3 is essential for polarization of tumor-associated neutrophils in non-small cell lung carcinoma

**DOI:** 10.1038/s41467-023-37515-8

**Published:** 2023-03-31

**Authors:** Jeff Yat-Fai Chung, Philip Chiu-Tsun Tang, Max Kam-Kwan Chan, Vivian Weiwen Xue, Xiao-Ru Huang, Calvin Sze-Hang Ng, Dongmei Zhang, Kam-Tong Leung, Chun-Kwok Wong, Tin-Lap Lee, Eric W-F Lam, David J. Nikolic-Paterson, Ka-Fai To, Hui-Yao Lan, Patrick Ming-Kuen Tang

**Affiliations:** 1 Department of Anatomical and Cellular Pathology, State Key Laboratory of Translational Oncology, The Chinese University of Hong Kong, Shatin, Hong Kong; 2 Guangdong Provincial Key Laboratory of Regional Immunity and Diseases, Carson International Cancer Center, Department of Pharmacology, Shenzhen University Health Science Center, Shenzhen, China; 3 Department of Medicine and Therapeutics, Li Ka Shing Institute of Health Sciences, The Chinese University of Hong Kong, Shatin, Hong Kong; 4 Department of Surgery, The Chinese University of Hong Kong, Shatin, Hong Kong; 5 College of Pharmacy, Jinan University, Guangzhou, China; 6 Department of Paediatrics, The Chinese University of Hong Kong, Shatin, Hong Kong; 7 Department of Chemical Pathology, The Chinese University of Hong Kong, Shatin, Hong Kong; 8 Reproduction, Development and Endocrinology Program, School of Biomedical Sciences, The Chinese University of Hong Kong, Shatin, Hong Kong; 9 Sun Yat-sen University Cancer Center, State Key Laboratory of Oncology in South China, Collaborative Innovation Center for Cancer Medicine, Guangdong, 510060 China; 10 Department of Nephrology and Monash University Department of Medicine, Monash Medical Centre, Clayton, VIC 3168 Australia

**Keywords:** Translational immunology, Translational research, Cancer microenvironment

## Abstract

Neutrophils are dynamic with their phenotype and function shaped by the microenvironment, such as the N1 antitumor and N2 pro-tumor states within the tumor microenvironment (TME), but its regulation remains undefined. Here we examine TGF-β1/Smad3 signaling in tumor-associated neutrophils (TANs) in non-small cell lung carcinoma (NSCLC) patients. Smad3 activation in N2 TANs is negatively correlate with the N1 population and patient survival. In experimental lung carcinoma, TANs switch from a predominant N2 state in wild-type mice to an N1 state in Smad3-KO mice which associate with enhanced neutrophil infiltration and tumor regression. Neutrophil depletion abrogates the N1 anticancer phenotype in Smad3-KO mice, while adoptive transfer of Smad3-KO neutrophils reproduces this protective effect in wild-type mice. Single-cell analysis uncovers a TAN subset showing a mature N1 phenotype in Smad3-KO TME, whereas wild-type TANs mainly retain an immature N2 state due to Smad3. Mechanistically, TME-induced Smad3 target genes related to cell fate determination to preserve the N2 state of TAN. Importantly, genetic deletion and pharmaceutical inhibition of Smad3 enhance the anticancer capacity of neutrophils against NSCLC via promoting their N1 maturation. Thus, our work suggests that Smad3 signaling in neutrophils may represent a therapeutic target for cancer immunotherapy.

## Abstract (web summary)

TGF-β stimulated tumor-associated neutrophils (TANs) can exert pro-tumoral functions. Here the authors show that Smad3 activation in TANs is associated with an N2-like polarization state and poor outcome in patients with non-small cell lung carcinoma and that Smad3 targeting reprograms TANs to an antitumor state suppressing tumor growth in preclinical lung cancer models.

## Introduction

Cancer remains a leading cause of death worldwide^1,2^. Cancer cells are heterogeneous, versatile, and adaptable, leading to primary and secondary resistance^3^. Indeed, only a small population of patients with advanced lung cancer respond to immunotherapy^4^. However, rather than targeting tumor cells directly, modifying the function of specfic cell types—particularly immune cells—within the tumor microenvironment (TME) is an alternative strategy since cancer growth, invasion, and metastasis depend upon stromal conditions^5^. Thus, a better understanding of the immunodynamics of the TME may uncover new strategies for the development of effective anticancer therapies.

Leukocytes make up a substantial component of stromal cells in lung cancer, with variable numbers of T cells, B cells, macrophages, dendritic cells, mast cells, and granulocytes present^6^. While the contribution of most of these immune cell types to tumor growth has been investigated in detail, the role of neutrophils is less well understood. Originally considered neutral bystanders, it is now evident that the TME can induce neutrophil polarization into different functional states^7^. Within the TME, neutrophils can be polarized to an antitumor N1 state or to a protumor N2 state^8^. However, the mechanisms regulating this polarization remain to be fully explored.

TGF-β is a potent immunosuppressive and differentiation factor that can promote tumor growth^9^. Treatment with a small molecule inhibitor of TGF-β receptor signaling substantially reduced the growth of a mouse mesothelioma tumor in association with increased CD8 + T cells^10^. This protective effect was also associated with increased tumor-associated neutrophils (TANs) and polarization of neutrophils to an antitumor N1 state^8,11^. Additional studies have shown that TGF-β can stimulate a protumor N2 neutrophil phenotype, but the underlying mechanisms remained unclear^12,13^. The TGF-β receptor complex can engage both Smad-dependent canonical signaling and Smad-independent non-canonical signaling^14,15^. Emerging evidence indicates the importance of Smad3 in lung cancer TME. Activation (phosphorylation) of SMAD3 in immune cells was negatively associated with the overall and disease-free survival of patients with lung cancer independent of their histological subtypes^16^. A critical role for canonical TGF-β/Smad3 signaling in cancer development was demonstrated by the substantial reduction tumor growth seen in *Smad3* gene-deficient mice (Smad3-KO)^17^. While TGF-β/Smad3 signaling suppresses natural killer cell (NK cell) activity and promotes the accumulation of cancer-associated fibroblasts^17,18^, the contribution of TGF-β/Smad3 signaling in the polarization of TANs and tumor growth remains unknown.

Here, we show that Smad3 activation in TANs is associated with a predominant N2 state of polarization and a poor outcome in patients with non-small-cell lung carcinoma (NSCLC). Smad3-KO mice exhibit increased neutrophil infiltration and a switch to a predominant N1 antitumor state in a lung cancer model. A functional role for these Smad3-KO N1 TANs in inhibiting tumor growth is confirmed by neutrophil depletion studies, while the adoptive transfer of Smad3-KO neutrophils inhibits tumor growth in wild-type mice. In addition, Smad3-KO neutrophils show the enhanced killing of tumor cells in vitro. Integrating analysis of the transcriptome and chromatin binding at the single cell level shows that Smad3 induces transcription of genes promoting a protumor N2 state in TANs, whereas, without Smad3, these TANs further differentiate into an antitumor N1 state. Thus, inhibition of Smad3 is identified as a therapeutic strategy to promote polarization of TANs to an antitumor N1 state and thus suppress the development of lung cancer.

## Results

### SMAD3 activation negatively correlates with N1 TAN abundance in NSCLC

**Fig. 1:**
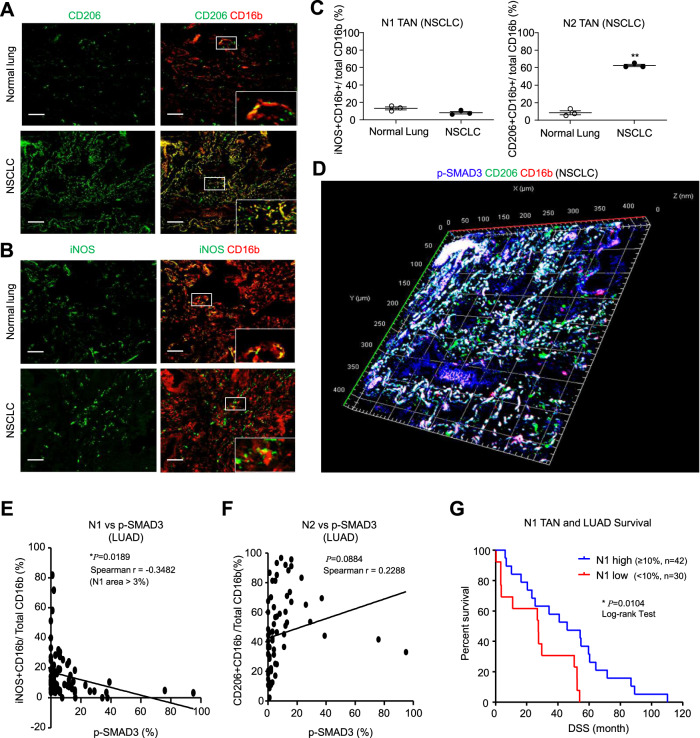
SMAD3 activation is negatively associated with an N1 phenotype in NSCLC. **A**–**C** Confocal imaging and quantification show a marked accumulation of N2 TANs (CD206^+^CD16b^+^ cells) in NSCLC compared to adjacent normal lung tissue, which contrasts with the small population of N1 TANs (iNOS^+^CD16b^+^ cells) (**P < 0.01 vs. normal lung; two-tailed *t*-test). **D** Three-dimensional confocal image showing SMAD3 activation (p-SMAD3, blue) in many N2 TANs (CD206^+^CD16b^+^ cells) in NSCLC. Correlation analysis shows that Smad3 activation (**E**) has a negative correlation with the proportion of N1 TANs (**F**) but does not correlate with the proportion of N2 TANs (Pearson correlation two-tailed test). **G** A low proportion of N1 TANs are significantly associated with higher patient mortality in NSCLC (log-rank (Mantel–Cox) test). Scale bar, 50 μm. **A**–**D** Data represents mean ± SEM of three patients/group. **E**–**G** Data based on a cohort of 72 patients. The exact *P* values of normal lung vs. NSCLC tissue are **1C**. *P* = 0.0655(N1 TAN) and *P* = 0.004(N2 TAN). Source data are provided as a Source Data file.

We investigated SMAD3 activation (phosphorylation) in TAN in a bank of NSCLC tissue samples. Neutrophils were identified by expression of the CD16b antigen^19–21^, which was validated in a fresh NSCLC sample by flow cytometry which demonstrated distinct CD16b^+^ neutrophil and CD68^+^ macrophage populations (Supplementary Fig. 1A). Multicolor immunostaining identified iNOS^+^CD16b^+^ and CD206^+^CD16b^+^ cells, designated as N1 and N2 TANs, respectively (Fig. 1A, B). N1 state TANs represented a low percentage of total CD16b^+^ cells in both NSCLC tumors and control lung tissue. By contrast, N2 state TANs represented over 50% of total CD16b^+^ cells in tumors, but less than 10% of CD16b^+^ cells in control lung tissue (Fig. 1C). Immunoperoxidase staining showed many p-SMAD3^+^ in tumors compared to few stained cells in control lung tissue (Supplementary Fig. 1B). Three-dimensional confocal imaging showed p-SMAD3 staining in many CD206^+^CD16b^+^ N2 TANs (Fig. 1D**)**. Unexpectedly, SMAD3 phosphorylation was negatively correlated with the percentage of N1 cells in total CD16b^+^ TANs but did not correlate with the percentage of N2 TANs in patients with lung adenocarcinoma in our patient cohort (*n* = 72, Fig. 1E, F and Supplementary Table 1). Based on the median value, a higher abundance of N1 TANs (≥10% of the area) was associated with better disease-free survival in NSCLC patients (Fig. 1G and Supplementary Data 1), whereas a lower N1/N2 or N1/p-SMAD3^+^ TAN ratio was associated with disease progression (Supplementary Fig. 1C, D). These findings demonstrate an association between SMAD3 activation and N1/N2 polarization in cancer progression.

### Smad3 deletion promotes N1 state TANs and reduces tumor growth in the LLC model

**Fig. 2:**
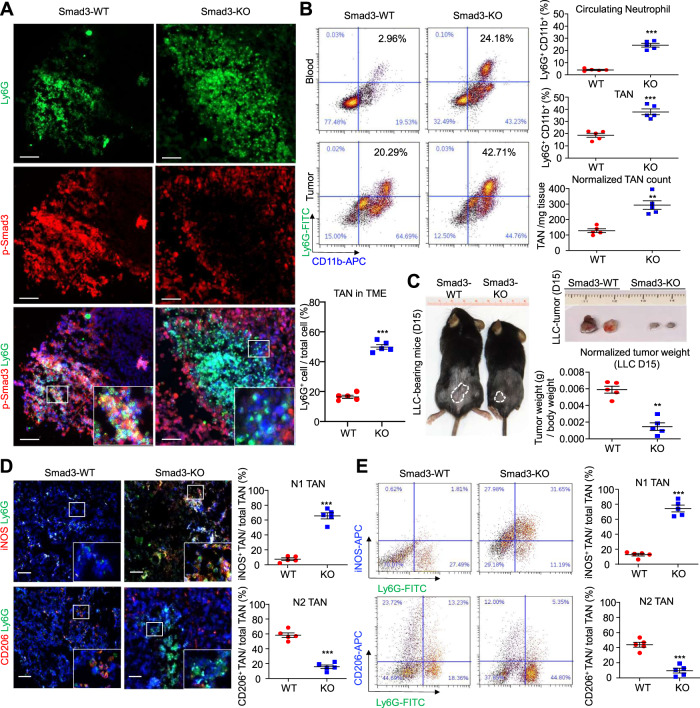
Smad3 deficiency increases the proportion of N1 TANs in LLC tumors. **A** Confocal imaging and **B** flow cytometry, show an increase in Ly6G^+^ neutrophils in the circulation and in the TME of LLC-bearing Smad3-KO mice compared to wild-type (Smad3-WT) controls (**P < 0.01, ***P < 0.001 vs. Smad3-WT, two-tailed *t*-test). **C** Smad3-KO mice show smaller tumors compared to Smad3-WT controls on day 15 (**P < 0.01 vs. WT, two-tailed *t*-test). **D** Immunofluorescence and **E** flow cytometric analysis showed a low proportion of N1 (iNOS^+^Ly6G^+^) and a high proportion of N2 (CD206^+^ Ly6G^+^) phenotype TANs in Smad3-WT mice, which is reversed in Smad3-KO mice which show a high abundance of N1 and a low abundance of N2 TANs (***P < 0.01 vs. WT, two-tailed *t*-test). Scale bars, 50 μm. **A**–**E** Data represents mean ± SEM of five mice/group. The exact *P* values of WT vs. KO are **2A**. *P* = 0.0002(TAN in TME), **2B**. *P* = 0.002(Circulating Neutrophil), *P* = 0.0004(TAN), *P* = 0.0078(Normalized TAN count). **2C**. *P* = 0.0011. **2D**. *P* = 0.0003(N1 TAN), *P* = 0.0002(N2 TAN). **2E**. *P* = 0.0002(N1 TAN), *P* = 0.0005(N2 TAN). Source data are provided as a Source Data file.

The role of Smad3 in regulating neutrophil recruitment and polarization in lung cancer was investigated in wild-type (Smad3-WT) and Smad3-knockout (Smad3-KO) mice using the syngeneic Lewis lung carcinoma (LLC) cell model^14,17^. LLC tumors in wild-type mice showed many p-Smad3^+^ cells, with two color staining showing numerous Ly6G^+^ neutrophils also exhibiting p-Smad3 staining (Fig. 2A and Supplementary Fig. 2A). Smad3-KO mice inoculated with LLC cells showed a marked increase in the abundance of neutrophils in both the circulation and the TME as shown by immunostaining and flow cytometry, as well as substantially less tumor growth on day 15 compared to wild-type controls (Fig. 2A–C and Supplementary Fig. 2A, B). While LLC tumors showed p-Smad3 staining in Smad3-KO mice (Fig. 2A), this staining was absent from Ly6G+ TANs and was attributed to Smad3 activation within LLC tumor cells (Supplementary Fig. 2A, B). LLC tumors in wild-type mice displayed a high proportion of TANs with an N2 phenotype (CD206^+^Ly6G^+^ cells) and low levels of TANs with an N1 phenotype (iNOS^+^Ly6G^+^ cells). By contrast, neutrophils in the TME in Smad3-KO mice contained a low proportion of N2 and a high proportion of N1 phenotype cells (Fig. 2D, E and Supplementary Fig. 2C, D), implying a role for Smad3 in the N1/N2 polarization of TAN in lung cancer.

### scRNA-seq analysis of Smad3 regulation of N1/N2 polarization in TANs

**Fig. 3:**
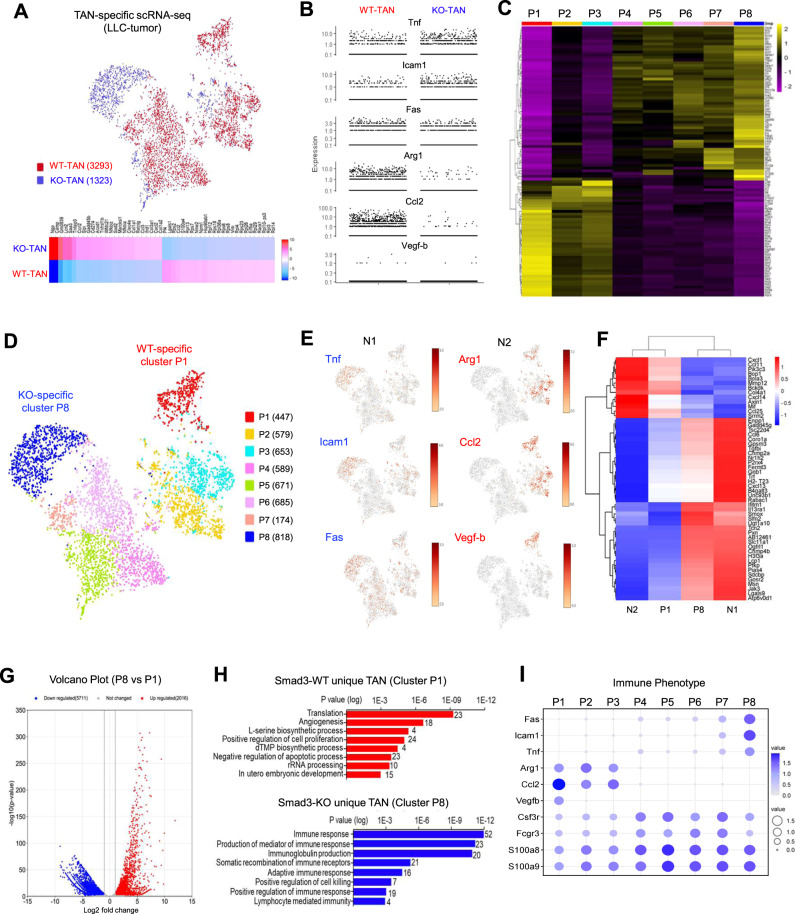
Transcriptome analysis showing that Smad3 regulates N1/N2 polarization in TANs. TANs (Ly6G^+^CD11b^+^ cells) sorted from LLC tumors in Smad3-WT and Smad3-KO mice (pooled from eight tumors /group) underwent scRNA-seq. **A** t-SNE plot and heatmap of the filtered scRNA-seq dataset show distinct transcriptome profiles of TANs from Smad3-WT (red) and Smad3-KO (blue) mice. **B** Expression plot shows Smad3-KO TANs predominantly express markers of an N1 phenotype (Tnf, Icam1, and Fas), whereas Smad3-WT TANs predominantly express markers of an N2 phenotype (Arg1, Ccl2, and Vegf-b). **C** Heatmap and **D** t-SNE plot showing the top 100 differential expressed genes (DEGs) and the relationship amongst eight clusters (P1 to P8) of Smad3-WT and -KO TANs unbiasedly grouped using the Louvain method. **E** t-SNE plots show enrichment of N2 markers (Arg1, Ccl2, and Vegf-b) in the Smad3-WT-specific P1 cluster and enrichment of N1 markers (Tnf, Icam1, and Fas) in the Smad3-KO-specific P8 cluster. **F** Heatmap analysis further indicates that P1 and P8 clusters share transcriptome signatures with N2 and N1 TANs, respectively, as defined by ref. ^23^. **G** Volcano plot shows the distinct transcriptome profile of P8 compared to P1 (statistical significance calculated by Loupe Cell Browser). **H** GO analysis of upregulated differentially expressed genes (DEGs) extracted from Smad3-WT (P1) and Smad3-KO (P8) specific clusters reveals that Smad3-KO TANs acquired anticancer functions via cell killing and immune responses, compared to Smad3-WT TANs which exhibit a protumor phenotype with functions of angiogenesis and positive regulation of cell proliferation. (Benjamini–Hochberg–corrected two-tailed *t*-test). **I** Dot plot visualizing the switch of N1 and N2 marker expression across clusters P1–P8, suggesting a regulatory role of Smad3 in the phenotypic shift of TANs.

To capture the transcriptome profile of TAN populations, we used Fluorescence-activated Cell Sorting (FACS) to isolate Ly6G^+^ CD11b^+^ neutrophils from LLC tumors dissected from Smad3-WT and Smad3-KO mice and performed 10X single-cell RNA sequencing (scRNA-seq)^14^ (Supplementary Fig. 3A). In our single-cell dataset, we sorted 4116 cells from Smad3-WT TANs and 2514 cells from Smad3-KO TANs with a median of 1311 unique molecular identifiers (UMIs) with 603 genes per cell (Supplementary Table 2). The dataset was then filtered according to multiple neutrophil-specific markers (S100a9, S100a8, Csf3r, and Il1rn)^22^, resulting in 3293 Smad3-WT TANs and 1323 Smad3-KO TANs retained for downstream analysis (Supplementary Fig. 3B). By merging the two cleaned datasets, we detected distinct transcriptome signatures of Smad3-WT (red) and Smad3-KO (blue) TANs (Fig. 3A, Supplementary Fig. 4A, and Supplementary Data 2), where N1 markers (*Tnf, Icam1,* and *Fas*) were highly expressed in the Smad3-KO clusters, whereas N2 markers (*Arg1, Ccl2*, *and Vegf-b*) were highly expressed in the Smad3-WT clusters (Fig. 3B). Smad3-WT and KO TANs were then unbiasedly clustered into P1 to P8 according to their transcriptome profile (Fig. 3C, D, Supplementary Fig. 4B, Supplementary Table 3, and Supplementary Data 2), where P1 is a WT-specific cluster with N2 marker expression and P8 is a KO-specific cluster with N1 markers expression as shown in t-SNE plots (Fig. 3E). By comparison with the reported gene signatures of N1 and N2 TANs^23^, we identified that the WT-specific cluster P1 corresponded to N2 TANs, whereas the KO-specific cluster P8 corresponded to N1 TANs (Fig. 3F and Supplementary Data 2). The upregulated differentially expressed genes (DEGs) of the Smad3-KO-specific cluster P8 were strongly associated with antitumor functions (immune response and positive regulation of cell killing), whereas protumor functions (angiogenesis and positive regulation of cell proliferation) were linked to the Smad3-WT-specific cluster P1 (Fig. 3G, H and Supplementary Data 2). Furthermore, we observed a progressive phenotypic change of N2 to N1 marker expression in clusters P1 to P8 (Fig. 3I), suggesting a regulatory role of Smad3 in neutrophil development.

### Smad3 limits neutrophil development in TME at the transcriptional level

**Fig. 4:**
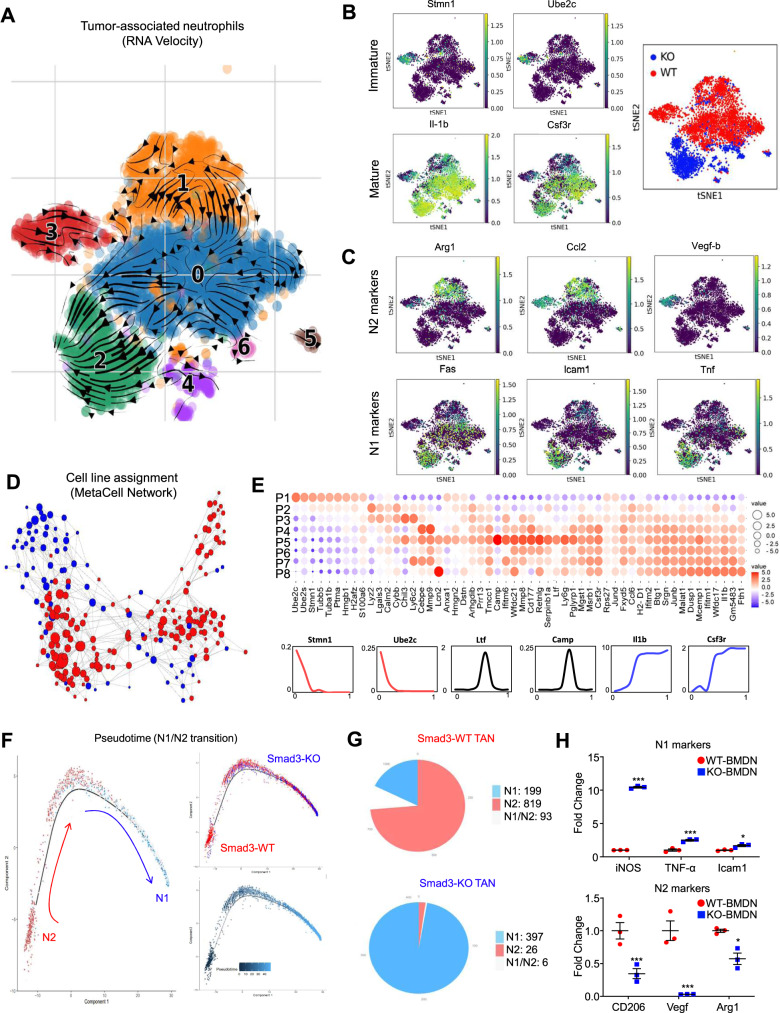
Transcriptome analysis shows a derivation of TAN development in N1/N2 TANs. **A** The arrows in the diffusion plot of RNA velocity analysis of the TAN dataset from Fig. 3A indicates that the Smad3-KO N1 population (cluster 2, green) is derived from the Smad3-WT N2 (cluster 3, red) as shown by: **B** expression of neutrophil maturation markers, **C** expression of N1/N2 markers, and **D** the cell line assignment (k-NN) MetaCell network graph showing the developmental relationship between Smad3-WT and KO TAN from Fig. 3A. **E** Dot plot showing the transcription gradient of neutrophil development gene expression from immature P1 to the mature P8 cluster. The size and color of the circles represent the value of log_2_ fold change and the direction of change in expression, respectively, of neutrophil development genes (lower panel) in each cluster. **F** Pseudotime analysis of the TAN scRNA-seq dataset clearly visualized an extended trajectory of the Smad-WT N2 (Ccl2^+^) phenotype towards the Smad3-WT N1 (Tnf^+^) phenotype within the LLC tumor, where **G** Smad3-KO TANs (397/429 cells = 92.5%) preserved an N1 phenotype compared to Smad3-WT cells (199/1111 cells = 17.9%). **H** RT-PCR analysis of BMDN stimulated LLC conditioned medium shows increased fold change of N1 markers (iNOS, Tnf-α, and Icam1) in Smad3-KO neutrophils, while Smad3-WT neutrophils showed increased levels of N2 markers (CD206, Vegf, and Arg1) (*P < 0.05, ***P < 0.001 vs. WT-BMDN, *n* = 3 independent samples, one-way ANOVA). **H** Data represents mean ± SEM from three independent experiments. The exact *P* values of WT-BMDN vs. KO-BMDN are **4H**. *P* = 0.0001 (iNOS), *P* = 0.0001(Tnf-α), *P* = 0.0149(Icam1), *P* = 0.001(CD206), *P* = 0.0001(Vegf), and *P* = 0.0211(Arg1). Source data are provided as a Source Data file.

To elucidate the potential regulatory role of Smad3 in neutrophil development in TME, we performed RNA velocity analysis to recapture the developmental pathway of Smad3-WT and -KO TANs in LLC tumor with single-cell resolution^24^. Unexpectedly, we found that Smad3-WT-specific TAN (cluster 3) can be gradually transited into Smad3-KO-specific TAN (cluster 2) indicated by their developmental direction arrows in Fig. 4A. Interestingly, we observed that N2 (*Arg1, Ccl2,* and *Vegf-b*) and N1 (*Fas, Icam1,* and *Tnf*) phenotypes were respectively associated with the expression levels of neutrophil immature (*Stmn1* and *Ube2c*) and mature (*Il-1b* and *Csf3r*) markers in TANs in a Smad3-dependent manner (Fig. 4B, C); suggesting that Smad3 may suppress N1 phenotype via limiting TAN maturation in TME. To validate the hypothesis, we examined the developmental pattern and potential connections of Smad3-WT and -KO TANs clusters in Fig. 3D with MetaCell pipeline^25^ and transcriptional signatures^26^. Encouragingly, we detected a strong developmental connection between Smad3-WT and -KO TANs (Fig. 4D) and a gradual maturation status from the Smad3-WT-specific cluster P1 to the Smad3-KO-specific cluster P8 at the transcriptional level (Fig. 4E and Supplementary Data 2). In addition, we characterized the intermediate clusters P2 to P7 according to their functions of mature neutrophils (e.g., chemotaxis, granule production, and ROS biosynthetic process^27^), revealing that P2-P3 belong to immature neutrophils with N2-like phenotype while P4-P7 showed a progressive maturation to a fully developed N1 phenotype (Fig. 3I, 4E and Supplementary Fig. 5A). Furthermore, we also confirmed the findings by reconstructing the developmental pathways of Smad3-WT and Smad3-KO TANs based on the expression levels of N1 (Tnf) and N2 (Ccl2) markers with pseudotime analysis (Fig. 4F), where additional N1/N2 markers and neutrophil development genes can be successfully mapped along the pseudotime pathway (Supplementary Fig. 5B, C). Thus, our findings discovered a developmental direction of TAN from N2 to N1 in TME, which can be blocked by the inhibitory role of Smad3 in neutrophil maturation.

In addition, we further validated our findings from the experimental lung carcinoma model with a public scRNA-seq dataset derived from patients with NSCLC to ensure its clinical relevance. The human TANs from the dataset were re-clustered through dimensionality reduction with UMAP and clustering based on the expression of marker genes (CSF3R, FPR1, NAMPT, MNDA, and FCGR3B) according to the 10x Genomics protocol (Supplementary Fig. 6A). Consistently, neutrophil immature markers (STMN1, UBE2S, and TUBA1B) were down-regulated in SMAD3-ve human TANs of NSCLC associated with their N1 signature (Supplementary Fig. 6B, C)^23^. In line with this notion, both genetic deletion and pharmacologic inhibition of Smad3 resulted in enhanced N1 development of mouse bone marrow-derived neutrophils (BMDNs) stimulated with LLC cancer cell conditioned medium (LLC-CM) in vitro, arguing that Smad3 restricts TAN maturation in lung cancer (Fig. 4H and Supplementary Fig. 7). Therefore, Smad3 may represent a potential therapeutic target for enhancing N1 anticancer immunity in lung cancer.

### Smad3 silencing enhances the anticancer activity of TAN in vivo and in vitro

**Fig. 5:**
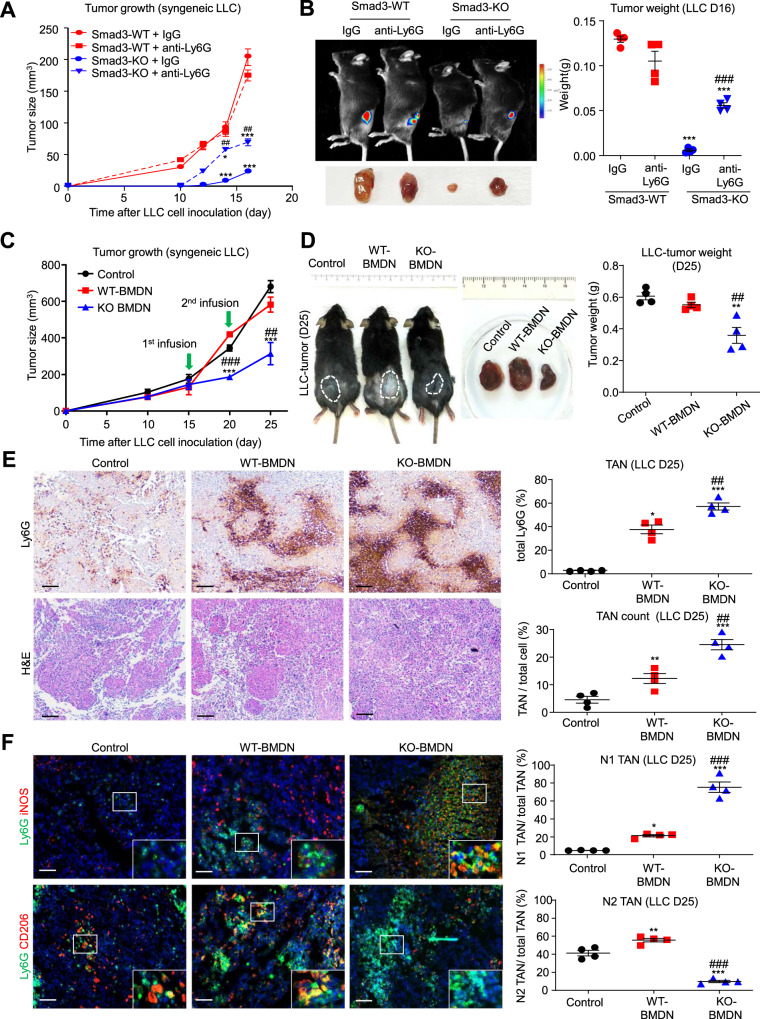
Silencing of Smad3 enhances the antitumor activity of TANs in vivo. **A**, **B** Smad3-WT and Smad3-KO mice were treated with anti-Ly6G or control IgG beginning 3 days before inoculation with LLC cells and killed on day 16 and analysed for; **A** tumor size, and **B** tumor weight (*P < 0.05, ***P < 0.001 vs. IgG-treated Smad3-WT mice; ##*P* < 0.01, ###*P* < 0.001 vs. IgG-treated Smad3-KO mice; one-way ANOVA). **C**–**F** Adoptive transfer of Smad3-knockout (KO-BMDN), but not wild-type BMDNs (WT-BMDN), on days 15 and 20 significantly inhibited the growth of LLC tumors in Smad3-WT mice as shown by: **C** tumor size, and **D** tumor weight, on day 25. **E** Immunohistochemistry and H&E staining shows that adoptive transfer of WT-BMDNs increases the proportion of neutrophils within the TME in LLC tumors, while the transfer of KO-BMDN resulted in an even greater proportion of neutrophils in the TME. **F** Immunostaining shows that the transfer of WT-BMDN did not affect the proportions of N1 and N2 TANs in the TME, whereas the transfer of KO-BMDN caused a substantial change in the proportion of N1 and N2 TANs. (*P < 0.05, **P < 0.01, ***P < 0.001 vs. Control, ##*P* < 0.01, ###*P* < 0.001 vs. WT-BMDN, one-way ANOVA). Scale bars, 50 μm. **A**–**F** Data represents mean ± SEM of 4 mice/group. The exact *P* values of IgG- vs. anti-Ly6G-treatment are **5A**. *P* = 0.0001 (IgG KO vs. IgG WT, D14), *P* = 0.0122 (anti-Ly6G KO vs. IgG WT, D14), *P* = 0.0049 (anti-Ly6G KO vs. IgG KO, D14), *P* = 0.0001 (IgG KO vs. IgG WT, D16), *P* = 0. 0001 (anti-Ly6G KO vs IgG WT, D16), *P* = 0.0085 (anti-Ly6G KO vs. IgG KO, D16), **5B**. *P* = 0.0001 (IgG KO vs IgG WT), *P* = 0.0001 (anti-Ly6G KO vs. IgG WT), and *P* = 0.0003 (anti-Ly6G KO vs. IgG KO). Exact *P* values of BMDN adoptive transfer are **5C**. *P* = 0.0001 (KO-BMDN vs. WT-BMDN, D20), *P* = 0.0001 (KO-BMDN vs. Control, D20), *P* = 0.007 (KO-BMDN vs. WT-BMDN, D25), *P* = 0.0009 (KO-BMDN vs. Control, D25). **5D**. *P* = 0.0068 (KO-BMDN vs. WT-BMDN), *P* = 0.0014 (KO-BMDN vs. Control), **5E**. *P* = 0.0013 (KO-BMDN vs. WT-BMDN, Ly6G), *P* = 0.0001 (KO-BMDN vs. Control, Ly6G), *P* = 0.0226 (WT-BMDN vs. Control, Ly6G), *P* = 0.0018 (KO-BMDN vs. WT-BMDN,H&E), *P* = 0.0001 (KO-BMDN vs. Control, H&E), *P* = 0.0001 (WT-BMDN vs. Control, H&E). **5F**. *P* = 0.0001 (KO-BMDN vs. WT-BMDN,N1 TAN), *P* = 0.0001 (KO-BMDN vs. Control, N1 TAN), *P* = 0.0205 (WT-BMDN vs. Control, N1 TAN), *P* = 0.0001 (KO-BMDN vs. WT-BMDN, N2 TAN), *P* = 0.0001 (KO-BMDN vs. Control, N2 TAN), *P* = 0.0039 (WT-BMDN vs. Control, N2 TAN). Source data are provided as a Source Data file.

We investigated the contribution of Smad3-WT and Smad3-KO neutrophils in LLC tumor growth by neutrophil depletion using the anti-Ly6G antibody (clone 1A8)^11,28^. Neutrophil depletion, which was validated by immunostaining in tumor tissues (Supplementary Fig. 8), resulted in a marked increase in tumor growth in Smad3-KO mice, but did not affect tumor growth in wild-type controls (Fig. 5A, B and Supplementary Fig. 8).

**Fig. 6:**
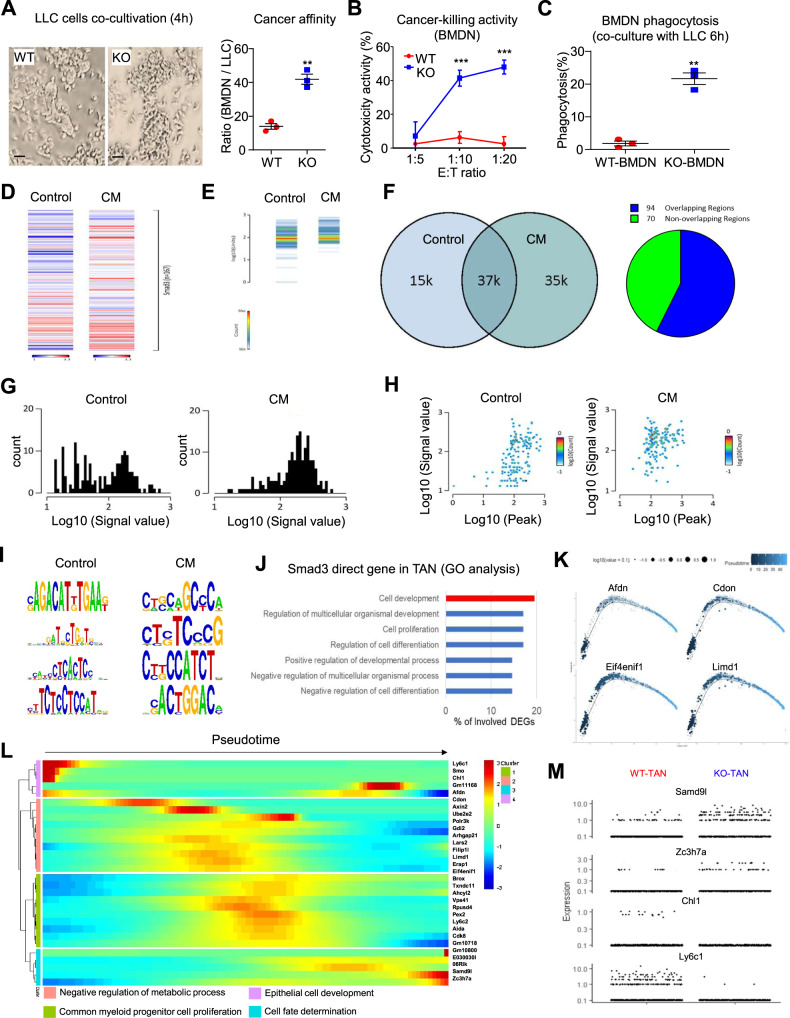
Smad3 regulates the development of TANs at the genomic level. **A**–**C** Smad3-WT and Smad3-KO-BMDN were co-cultured with LLC cells and assayed for; **A** binding of BMDN to LLC cells, **B** killing of LLC cells at different ratios of BMDN to LLC cells, and **C** phagocytosis of Dil-labeled LLC cells (**P < 0.01, ***P < 0.001 vs. WT-BMDN, *n* = 3 independent samples, two-tailed *t*-test) respectively. **D**–**J** Smad3-WT-BMDN (pooled from 4 mice /group) were cultured with 10% LLC-CM or normal media (control) for 2 h and analysed by Smad3-specific ChIP-seq. A substantial change in Smad3 binding to the genome was evident in BMDN stimulated by LLC-CM as shown by; **D**, **E** heatmap analysis, **F** overlap region chart, **G** enrichment plot, **H** correlation plot, and **I** binding motif analysis. **J** Gene ontology (GO) annotation reveals Smad3 directly regulates genes associated with cell development and differentiation in BMDNs stimulated with LLC-CM. Expression profiles of Smad3 target genes (**K**) related to neutrophil development (Afdn, Cdon, Elf4enif1, and Limd1) (statistical significance calculated from Monocle package) and **L** their functional annotation along the developmental pathway TANs (as in Fig. 3I). **M** Expression plot of N1 (Smad9l and Zc3h7a) and N2 (Chl1 and Ly6c1) Smad3 target genes in Smad3-WT and Smad3-KO TANs. Scale bar, 50 μm. **A**–**C** Data represents mean ± SEM from three independent experiments. The exact *P* values of WT-BMDN vs. KO-BMDN are **6A**. *P* = 0.0021. **6B**. *P* = 0.0001(1:10), *P* = 0.0001(1:20). **6C**. *P* = 0.003. Source data are provided as a Source Data file.

An adoptive transfer approach was used as an additional strategy to examine how Smad3-KO and Smad3-WT neutrophils can affect tumor growth. Wild-type mice with matched tumor sizes were injected on days 15 and 20 with MACS-sorted Smad3-WT or Smad3-KO BMDNs, and tumors were assessed on day 25 (Supplementary Fig. 9). The transfer of wild-type BMDN did not affect tumor growth despite an increased number of TANs being evident, although the N1/N2 balance of TANs was not affected (Fig. 5C–F). By contrast, the adoptive transfer of Smad3-KO-BMDN caused a substantial reduction in tumor growth in association with an even greater increase in total TANs, which exhibited a dominant N1 phenotype (Fig. 5C–F). Enhanced recruitment of Smad3-KO-BMDN over that of Smad3-WT-BMDN into the TME was demonstrated in an adoptive transfer experiment in which equal numbers of dye-labeled Smad3-WT and Smad3-KO-BMDN were injected into tumor-bearing mice, and the TME examined 24 h later (Supplementary Fig. 10). Finally, we assayed the antitumor activity of Smad3-WT and Smad3-KO-BMDN in co-culture with LLC cells. Smad3-KO-BMDN exhibited increased binding to LLC cells, much greater cytotoxicity towards LLC cells, and increased phagocytosis of LLC cells compared to Smad3-WT-BMDN (Fig. 6A–C and Supplementary Fig. 11). In sum, these findings identify Smad3 as an immunoregulator that facilitates tumor growth via polarizing neutrophils towards an N2 phenotype within the TME.

### Smad3 regulates the development of TANs at the genomic level

Smad3 is a transcription factor that facilitates some of the pathological actions of TGF-β1 in disease^14,29^. Therefore, we investigated the gene promoters to which Smad3 binds in neutrophils at the genomic level. BMDN isolated from wild-type mice were cultured for 2 h with or without LLC cell conditioned medium (LLC-CM) and then assayed by ChIP-sequencing. Compared to control cells, LLC-CM stimulation markedly altered the Smad3 binding pattern in the genome of BMDN (Fig. 6D, H). The Smad3 binding motifs were mapped to the genome of BMDN under control and LLC-CM stimulation conditions. This identified 64 direct Smad3 target genes under LLC-CM stimulation, including candidate genes responsible for cell development and differentiation (Fig. 6I, J and Supplementary Data 3). Of note, we identified several neutrophil N2 developmental factors (such as Afdn^30^, Cdon^31^, Elf4enif1^32^, and Limd1^33^) as Smad3 target genes under LLC-CM stimulation conditions (Fig. 6K). Moreover, interrogating the developmental pathway reconstructed from the TAN scRNA-seq dataset with the Smad3 target genes identified by ChIP-seq, identified enrichment of these genes in the N2 state (Fig. 6L). By comparing total Smad3-WT and Smad3-KO TANs, we confirmed that expression levels of Smad3 target genes by ChIP-seq were associated with N1 and N2 states that operate in a Smad3-dependent fashion (Fig. 6M). These findings highlight the regulatory role of Smad3 in N1/N2 polarization of TANs in lung carcinoma.

### Targeting SMAD3 enhances neutrophil cytotoxicity in human lung tumor cells

**Fig. 7:**
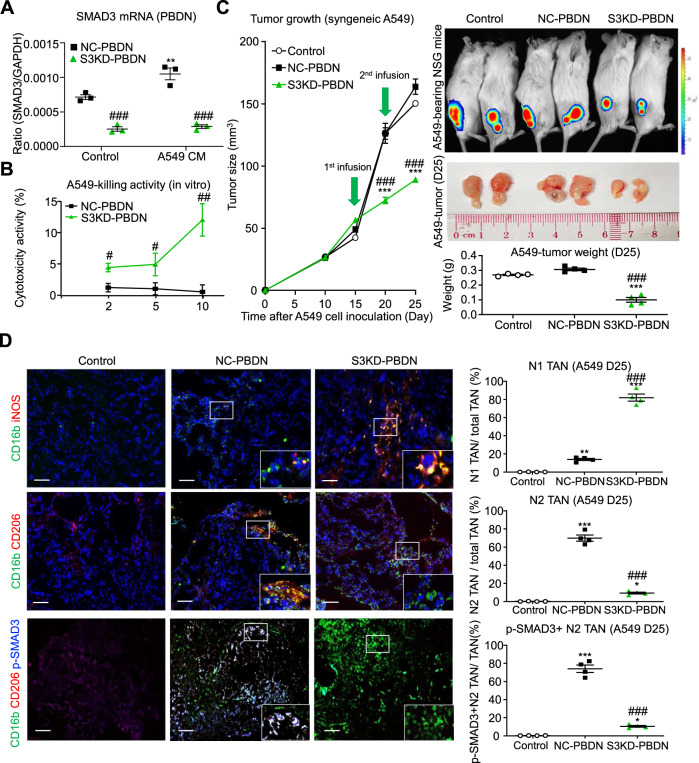
Neutrophil-specific silencing of SMAD3 enhances an N1 phenotype in NSCLC. **A** RT-PCR analysis showing that transfection of human PBDN with SMAD3 siRNA (S3KD-PBDN), but not with nonsense control siRNA (NC-PBDN), suppresses basal (control) and A549 conditioned medium (A549 CM) stimulated to increase in SMAD3 expression (**P < 0.01 vs. Control, ###*P* < 0.001 vs. NC-PBDN, *n* = 3 independent samples, one-way ANOVA). **B** Co-culture studies show enhanced cytotoxicity of S3KD-PBDN towards the human NSCLC cell line A549 (**P < 0.01, ***P < 0.001, *n* = 3 independent samples, two-tailed *t*-test). **C**, **D** NSG immunocompromised mice were inoculated with A549 cells and then received injections of human NC-PBDN or S3KD-PBDN on days 15 and 20 and then killed on day 25. **C** Adoptive transfer of S3KD-PBDN caused a significant reduction in tumor size and weight, which was not apparent with a transfer of NC-PBDN (***P < 0.001 vs. untreated A549 tumors (Control), ###*P* < 0.001 vs. NC-PBDN, one-way ANOVA). **D** Immunostaining for human neutrophil markers showed that adoptive transfer of NC-PBDN resulted in a high proportion of TANs exhibiting an N2 phenotype (CD206^+^ CD16b^+^ cells), whereas transfer of S3KD-PBDN resulted in a high proportion of TANs exhibiting an N1 phenotype (iNOS^+^ CD16b^+^ cells). Furthermore, p-SMAD3 staining was seen in a high proportion of N2, but not in N1 TANs. (*P < 0.05, **P < 0.01, ***P < 0.001 vs. untreated A549 tumors (Control), ###*P* < 0.001 vs. NC-PBDN, one-way ANOVA) Scale bars, 50 μm. **A**, **B** Data represents mean ± SEM from three independent experiments. **C**, **D** Data represents mean ± SEM of 4 mice/group. The exact *P* values of NC-PBDN vs. S3KD-PBDN are **7A**. *P* = 0.001 (Control), *P* = 0.0001(A549 CM-treated), *P* = 0.007 (NC-PBDN, Control vs. A549 CM-treated). **7B**. *P* = 0.017 (1:2), *P* = 0.0157 (1:5), *P* = 0.0077 (1:10). Exact *P* values of PBDN treatment are **7C**. *P* = 0.0002 (S3KD-PBDN vs. NC-PBDN, D20), *P* = 0.0002 (S3KD-PBDN vs Control, D20), *P* = 0.0001 (S3KD-PBDN vs. NC-PBDN, D25), *P* = 0.0001 (S3KD-PBDN vs. Control, D25), *P* = 0.0001 (S3KD-PBDN vs. NC-PBDN, tumor weight), *P* = 0.0001 (S3KD-PBDN vs. Control, tumor weight). **7D**. *P* = 0.0001 (S3KD-PBDN vs. NC-PBDN, N1 TAN), *P* = 0.0001 (S3KD-PBDN vs. Control, N1 TAN), *P* = 0.0068 (NC-PBDN vs. Control, N1 TAN), *P* = 0.0001 (S3KD-PBDN vs. NC-PBDN, N2 TAN), *P* = 0.0404 (S3KD-PBDN vs. Control, N2 TAN), *P* = 0.0001 (NC-PBDN vs. Control, N2 TAN), *P* = 0.0001 (S3KD-PBDN vs. NC-PBDN, SMAD3 + N2 TAN), *P* = 0.0398 (S3KD-PBDN vs. Control, SMAD3 + N2 TAN), *P* = 0.0001 (NC-PBDN vs. Control, SMAD3 + N2 TAN). Source data are provided as a Source Data file.

We next examined whether SMAD3 regulates N1/N2 polarization and tumor killing in human neutrophils in vitro and in vivo using the human NSCLC cell line, A549. First, we silenced SMAD3 expression in human peripheral blood-derived neutrophils (PBDNs) by siRNA transfection and evaluated their anticancer activity against A549 cells in vitro. As shown in Fig. 7A, B, silencing of SMAD3 in PBDN (S3KD-PBDN) significantly enhanced their ability to kill A549 cells in vitro, compared to the nonsense-treated control PBDN (NC-PBDN). Moreover, adoptive transfer of the S3KD-PBDNs into A549-bearing NSG mice caused a significant inhibition of tumor growth in vivo, whereas transfer of NC-PBDN were without effect (Fig. 7C). In contrast to the predominant N2 polarization (CD206^+^CD16b^+^ cells) with SMAD3 activation of TAN in the mice receiving NC-PBDN, there was a predominant N1 polarization (iNOS^+^CD16b^+^ cells) and lack of SMAD3 activation in TAN of mice receiving S3KD-PBDN (Fig. 7D). These findings show that inhibition of SMAD3 in human neutrophils promotes an N1 polarization within the TME and an antitumor function.

**Fig. 8:**
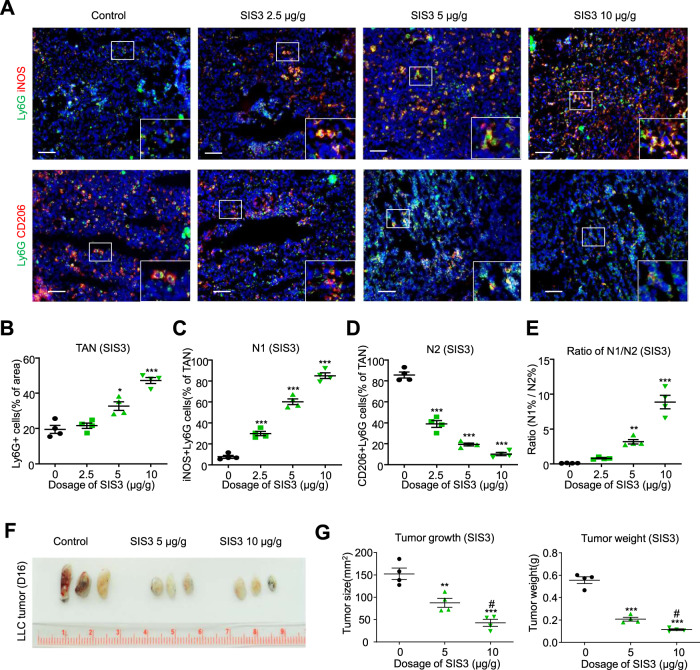
Pharmaceutical inhibition of Smad3 promotes an N1 phenotype and suppresses tumor growth. On day 7, after inoculation with LLC cells, Smad3-WT mice received daily administration of different doses of the Smad3 inhibitor, SIS3, or vehicle, until mice were killed on day 16. Immunostaining shows that SIS3 treatment increased the proportion of neutrophils (Ly6G^+^ cells) within the TME (**A**, **B**), increased the proportion of N1 (iNOS^+^ Ly6G^+^) TANs (**C**), decreased the proportion of N2 (CD206^+^ Ly6G^+^) TANs (**D**), changed the ratio of the N1/N2 phenotype in TANs (**E**), and suppressed LLC tumor growth and weight (**F**, **G**). (*P < 0.05, **P < 0.01, ***P < 0.001 vs. vehicle control, #*P* < 0.05, ##*P* < 0.01 vs. SIS3 5 μg/g, one-way ANOVA). Scale bars, 50 μm. **A**–**G** Data represents mean ± SEM of 4 mice/group. The exact *P* values of treatment vs. control are **8B**. *P* = 0.016 (SIS3 5 μg/g vs. vehicle), *P* = 0.0001 (SIS3 10 μg/g vs. vehicle)**, 8C**. *P* = 0.0001 (SIS3 2.5, 5, 10 μg/g vs. vehicle). **8D**. *P* = 0.0001 (SIS3 2.5, 5, 10 μg/g vs. vehicle). **8E**. *P* = 0.029 (SIS3 5 μg/g vs. vehicle), *P* = 0.0001 (SIS3 10 μg/g vs. vehicle). **8G**. *P* = 0.0042 (SIS3 5 μg/g vs. vehicle, tumor size), *P* = 0.0001 (SIS3 10 μg/g vs. vehicle, tumor size), *P* = 0.0335 (SIS3 5 μg/g vs. SIS3 10 μg/g, tumor size), *P* = 0.0042 (SIS3 5 μg/g vs. vehicle, tumor weight), *P* = 0.0001 (SIS3 10 μg/g vs. vehicle, tumor weight), *P* = 0.0273 (SIS3 5 μg/g vs. SIS3 10 μg/g, tumor weight). Source data are provided as a Source Data file.

Finally, we asked whether a drug-based approach could be used to promote N1 polarization of TANs and reduce lung tumor growth. Wild-type mice were given daily treatment with varying doses of the Smad3 inhibitor, SIS3, or vehicle, beginning on day 7 after inoculation with LLC cells. Compared to vehicle treatment, SIS3 treatment showed a dose-response in terms of reducing tumor size and weight, increasing the total number of TANs, and inducing N1 polarization to markedly change the N1/N2 ratio (Fig. 8A–G). Taken together, our results identify Smad3 as a regulator of neutrophil N1/N2 polarization within the TME and demonstrate that Smad3 is a druggable target to enhance neutrophil-mediated antitumor activity in lung cancer.

## Discussion

Neutrophils have long been known to play a key role in response to infectious organisms, whereas their role in cancer development has only recently become an area of great interest^7,34^. In this study, by resolving the transcriptome dynamics of neutrophils in the TME at a single-cell resolution, we demonstrate that Smad3 induces polarization of TAN to a predominant N2 state in lung cancer, whereas deletion of Smad3 induces polarization to an N1 state. This profound difference in N1/N2 polarization in wild-type versus Smad3-KO TANs was functionally important, as shown by the potent antitumor activity of Smad3-KO TANs.

Fridlender et al. suggested the terms N1 and N2 to represent antitumor and protumor neutrophil phenotypes, respectively, akin to the M1 and M2 phenotypes of tumor-associated macrophages^11,35^. Subsequent studies identified *Tnf*, *Icam1*, and *iNOS* as signature genes for the N1 phenotype, with *Vegf-b*, *Arginase*, and *Cd206* as signature genes for the N2 phenotype^23,36,37^. Notably, single antigen markers are not able to uniquely identify N1 or N2 neutrophil populations; this requires the use of a neutrophil-specific marker in combination with one or more additional markers. Therefore, in mouse studies, we used double staining for iNOS/Ly6G to detect N1 state TANs and CD206/Ly6G staining for N2 state TANs. In studies of human NSCLC, we employed iNOS/CD16b and CD206/CD16b double staining to identify N1 and N2 TANs, respectively. While wild-type mice with LLC tumors showed a predominant N2 phenotype in the TME, Smad3-KO mice showed an opposite polarization with a predominant N1 phenotype in TANs. In human studies, CD16b is considered a specific neutrophil marker^19–21^. However, it is important to acknowledge that immune cells can change their marker expression during an immune response. Therefore, we used flow cytometry to validate that CD16b is expressed by neutrophils, but not by CD68 + monocyte/macrophages, in the TME of human NSCLC. Thus, one important finding of this study is a validation of CD16b/iNOS and CD16b/CD206 as markers to identify human N1 and N2 TANs, respectively, using flow cytometry or confocal microscopy.

The function of TGF-β/Smad3 signaling in cancer is highly context-dependent, with both cancer-promoting and cancer-suppressing effects described in ref. ^38^. A biopsy study of early-stage NSCLC found that SMAD3 phosphorylation in T cell populations and in macrophages in the TME negatively impacted overall and partially disease-free survival, although SMAD3 activation in neutrophils was not assessed^16^. Our biopsy study in NSCLC extends these findings by demonstrating that SMAD3 activation is prominent in N2 TANs, that SMAD3 activation negatively correlates with the percentage of N1 state TANs, and that a greater percentage of N1 TANs in the TME is associated with better disease-free survival.

We previously showed that Smad3-KO mice exhibit a profound reduction in tumor growth using the LLC and B16F10 cancer cell lines. Importantly, the deletion of Smad3 in only the bone marrow compartment was sufficient to provide this protection^17,39^. Here, we show that Smad3-KO neutrophils polarize to an N1 state with a potent antitumor effect in the syngeneic LLC lung cancer model. Neutrophil depletion in Smad3-KO mice abolished protection against lung tumor growth, establishing the antitumor role of Smad3-KO neutrophils. By contrast, neutrophil depletion in wild-type mice had no effect on tumor growth, arguing that despite a predominant N2 TAN protumor phenotype, they did not significantly impact tumor development. Indeed, the use of neutrophil depletion in mouse models of lung cancer have produced disparate results, with some showing antitumor effects^11,37^, some showing no effect^40,41^, and others showing enhanced growth and metastasis as a result of neutrophil depletion^42,43^. To further substantiate the function of N1 Smad3-KO neutrophils in lung cancer development, we performed adoptive transfer studies, which showed that delivery of Smad3-KO-BMDN, but not wild-type BMDN, could suppress tumor growth. This finding was confirmed in immunocompromised mice with the A549 cell tumor in which adoptive transfer of human PBDN with SMAD3 knock-down suppressed tumor growth, while control PBDN had no effect. In addition, deletion or knock-down of Smad3 in mouse or human neutrophils augmented their cytotoxic response to tumor cells in vitro. Together, these independent experiments point to Smad3 driving a protumor N2 state in TANs, while Smad3 deletion/knock-down exposes an alternative N1 state in TANs with potent antitumor activity.

The application of single-cell RNA-seq provides important insights into Smad3 regulation of N1/N2 polarization in TANs. The directed information of TAN maturation regarding N1/N2 phenotypes was demonstrated by RNA velocity and further resolved with MetaCell analysis via intensive analysis of clusters according to gene signatures of neutrophil development and functions^26,27^. We observed that N2 is an early state of TANs which progressively develops towards an N1 state in the TME. By using the expression pattern of signature genes identifying the N1 state (*Tnf*, *Fas*), the N2 state (*Arg1*, *Ccl2*), and immature neutrophils (*Stmn1*, *Ube2c*), pseudotime analysis confirmed the developmental direction of TANs with N2 as an early state of TANs in the lung cancer TME. We also validated that our findings can be applied to human TANs by interrogation of an NSCLC scRNA-seq dataset. Furthermore, we identified that Smad3 deficiency drives the polarization of TANs to an N1 state at the transcriptome level. Consistent with this finding, the culture of Smad3-KO-BMDN under stimulation by tumor cell conditioned media resulted in a predominant N1 phenotype, whereas wild-type BMDN polarized into a predominant N2 phenotype. In addition, mechanisms by which Smad3 regulates the polarization of BMDN stimulated with tumor cell cultured media was investigated at the genome level via ChIP-seq and unbiased bioinformatic analysis. Gene ontology analysis showed that direct targets of Smad3 in the stimulated BMDN are associated with cell development and differentiation. Moreover, we also confirmed the enrichment of these target genes along the pseudotime analysis of the development of the N2 state. Some of those highlighted Smad3 target genes have been reported to relate to neutrophil development and function; for example, Elf4ebp1 has been implicated in neutrophil differentiation, albeit in the HL-60 cell line^44^. Further studies are required to investigate how these Smad3 target genes contribute to N2 polarization in the TME. Taken together, these data identify Smad3 as a key regulator of the N1/N2 polarization of TANs operating at the level of the genome and transcriptome.

It is known that TGF-β/Smad3 signaling in leukocytes promotes tumor growth by suppressing antitumor cytotoxicity by CD8 + T cells^10^ and NK cells^9^, and by promoting the transition of tumor-associated macrophages into fibroblasts within the TME^14^. Here we demonstrate that TGF-β/Smad3 signaling also promotes tumor growth by promoting a protumor N2 state in TANs. Notably, while the suppressive effect of Smad3 signaling on NK cell antitumor activity can only be demonstrated in models with depletion of background immunity^9^, here we show that Smad3-KO BMDNs are effective in suppressing cancer progression in the immunocompetent TME. Another interesting finding was that Smad3 deletion led to increased neutrophil recruitment into the TME. This was evident in both the Smad3-KO mice, and with an increased number of TANs following the adoptive transfer of Smad3-KO versus Smad3-WT-BMDN in the LLC cancer model. Furthermore, using dye labeling we demonstrated that Smad3-KO-BMDN shows enhanced infiltration into the TME of wild-type mice, arguing that this enhanced recruitment is not due to factors produced by the TME, but rather a difference in the response of the Smad3-KO neutrophils per se. This increased infiltration of Smad3-KO neutrophils in the TME is consistent with the increased neutrophil infiltration seen in Smad3-KO mice in models of skin contact hypersensitivity and cutaneous irradiation^45,46^. Furthermore, given the interest in neutrophils for the tumor-specific delivery of anticancer drugs^47,48^, then utilizing Smad3-KO neutrophils might aid in this effort.

Targeting TGF-β signaling in cancer has moved into clinical trials with an inhibitor of the kinase activity of the TGF-β receptor type I (TGFBR1/ALK5), Vactosertib, now in several trials, including a phase 2 trial in NSCLC (NCT04515979). This builds on the work of ref. ^11^, which showed that oral administration of the TGFBR1 kinase inhibitor, SM16, suppressed the growth of a mouse lung tumor in association with a polarization of neutrophils to an antitumor N1 state. Theoretically, targeting SMAD3 is expected to cause fewer side effects than targeting TGFBR1, which would allow for a greater degree of SMAD3 inhibition compared to the tolerable level of TGFBR1 inhibition. We have demonstrated a highly effective suppression of LLC lung cancer growth using the Smad3 inhibitor, SIS3, which was associated with increased numbers of TANs and polarization of TANs to a predominant N1 state. Further studies are warranted to compare optimal Tgfbr1 inhibition versus optimal Smad3 inhibition in the LLC tumor model, and whether a Tgfbr1 inhibitor provides any added benefit when given to Smad3-KO mice. Given that more soluble forms of the SIS3 compound have been produced^49^, this might be a fruitful area for further investigation.

In summary, in this study, we define the role of Smad3 in the polarization of neutrophils within the tumor microenvironment. While Smad3 signaling promotes a protumor N2 state in TANs, the lack of Smad3 in neutrophil results in the opposite outcome, with TANs polarization into an N1 state with a potent antitumor activity based on increased recruitment and increased tumor cytotoxicity. These insights may have relevance for developing neutrophil-based immunotherapy against NSCLC.

## Methods

### Antibodies

Monoclonal antibodies used for immunostaining and flow cytometry included; mCD206-PE (1:100, 141706, BioLegend, clone:C068C2), hCD206 (1:100, sc-376232, Santa Cruz,clone:C-10),hCD206-FITC(1:100, 321104, BioLegend, clone:15C2), iNOS (1:100, sc-7271, Santa Cruz,clone:C-11), TNF-α (1:100, sc-52746, Santa Cruz,clone:C-4), Icam1 (1:100, sc-8439, Santa Cruz,clone:G-5), p-Smad3 (paraffin section, 1:100, sc-517575, Santa Cruz,clone:1D9), CD16b-PE (1:100, 550868, BD Biosciences, clone:CLB-gran11.5), Ly6G-FITC (1:100, 127606, BioLegend,clone:1A8), Ly6G-PE (1:100, 127608, BioLegend,clone:1A8), Ly6G-APC (1:100, 127614, BioLegend,clone:1A8), p-Smad3 (1:200, 600-401-919, Rockland, Polyclonal), CD11b-APC (1:100, 553311, BD Biosciences,clone:M1/70 (RUO)). CD68-APC(1:100, 333810, BioLegend, clone:Y1/82 A). Antibodies used for ChIP assay included anti-Smad3 (1:100, 9523 S, Cell Signaling Technology, clone:C67H9) and IgG Isotype Control (1:1000, 3900S, Cell Signaling Technology, clone: DA1E).

The polymer and HRP conjugated secondary antibodies used included EnVision+system-HRp-labeled Polymer Anti-mouse (100 µL per section, K4003, Dako), EnVision+system-HRp-labeled polymer anti-rabbit(100 µL per section, K4003, Dako), Goat anti-Rat IgG (H + L) Secondary Antibody, HRP(1:200,31470, Thermo fisher). Alexa Fluor conjugated secondary antibodies used included Goat anti-Mouse IgG (H + L) Cross-Adsorbed Secondary Antibody, Alexa Fluor™ 488(A-11001, Invitrogen), Goat anti-Mouse IgG (H + L) Highly Cross-Adsorbed Secondary Antibody, Alexa Fluor™ 546(A-11035, Invitrogen), Goat anti-Rabbit IgG (H + L) Highly Cross-Adsorbed Secondary Antibody, Alexa Fluor™ 546(A-11035, Invitrogen), Goat anti-Rabbit IgG (H + L) Highly Cross-Adsorbed Secondary Antibody, Alexa Fluor™ Plus 647(A32733, Invitrogen), Goat anti-Mouse IgG (H + L) Highly Cross-Adsorbed Secondary Antibody, Alexa Fluor™ Plus 647(A32728, Invitrogen)

### Patient samples and buffy coat

Paraffin sections of lung adenocarcinoma and of macroscopically normal lung parenchyma at least 5 cm away from the tumor in the same patient, were collected from the Prince of Wales Hospital, Chinese University of Hong Kong, Hong Kong. The healthy human buffy coats were supplied by Hong Kong Red Cross Blood Transfusion Service. The study was conducted according to the principles of the Declaration of Helsinki. Written informed consent was obtained from all patients and buffy coat donors. This study was approved by the Joint Chinese University of Hong Kong—New Territories East Cluster Clinical Research Ethics Committee (CREC Ref. No.: 2018.054).

### Mouse tumor models

All experimental procedures were approved by the Animal Experimentation Ethics Committee of the Chinese University of Hong Kong (CUHK) (AEEC Ref No.: 18/005/GRF) and were carried out in accordance with the Guide for the Care and Use of Laboratory Animals. All mice were kept by the Laboratory Animal Service Center of CUHK and maintained at 22–23 °C, <70% relative humidity with an alternating 12 h light/dark cycle with free access to standard mouse diet and water. Smad3-wild-type (Smad3-WT), Smad3-deficient (Smad3-KO) mice on C57BL/6 J background (both sexes, aged 8–12 weeks) and NOD.Cg-Prkdc^scid^Il2rg^tm1Wjl^/SzJ mice (NSG mice, male, aged 8–10 weeks) were used in this study. Smad3-KO mice (exon 8 deleted and exon 7 disrupted) were kindly provided by Dr. Chuxia Deng^50^. NSG mice were purchased from CUHK Laboratory Animal Services Centre. Syngeneic LLC tumors were induced by subcutaneous injection of 2 × 10^6^ LLC cells into the right flanks of Smad3-WT and Smad3-KO mice as described in our previous study^17^. A549 human lung cancer xenograft model was induced by subcutaneous injection of 2 × 10^6^ A549 cells into the right flanks of NSG mice as previously reported^17^. The maximal tumor size of 2000 mm^3^ was permitted by AEEC. In some cases, this limit has been exceeded by the last day of measurement and the mice were immediately euthanized.

### Lung carcinoma cell lines

Lewis lung carcinoma LLC (CRL-1642, ATCC) cells were cultured in Dulbecco’s Modified Eagle Medium/Nutrient Mixture F-12 medium (DMEM/F12) with 10% heat-inactivated fetal bovine serum (FBS) (Gibco), 100 U/ml penicillin G, and 100 mg/ml streptomycin. Human lung carcinoma A549 (CCL-185, ATCC) cells were cultured in Roswell Park Memorial Institute1640 medium (RPMI 1640) with 5% heat-inactivated FBS (Gibco), 100 U/ml penicillin G, and 100 mg/ml streptomycin. Cancer cell conditioned medium was collected after overnight incubation of LLC cells with serum-free DMEM/F12 (LLC-CM) or A549 cells with serum-free RPMI 1640 (A549 CM), followed by filtration with 0.2-μm nylon membranes.

### Tyramide signal amplification-based immunofluorescent multiplexing (Opal) and Immunohistochemistry

Formalin-fixed paraffin-embedded tissues (FFPE) and tumor microarray (TMA) sections (5 µm thickness) were deparaffinized in xylene and rehydrated in descending alcohols, followed by blocking of endogenous peroxidase in 3% hydrogen peroxide solution (30 min) and heat-induced epitope retrieval in citrate buffer (95 °C, 5 min). Sections were incubated with CD206 (1:100, Clone: C-10, Santa Cruz), iNOS (1:100, Clone: C-11, Santa Cruz), CD16b (1:100, Clone: CLB-gran11.5, BD Biosciences), Ly6G (1:100, Clone: 1A8, BioLegend), p-Smad3 (1:100, Clone: 1D9, Santa Cruz), p-Smad3 (1:200, 600-401-919, Rockland) antibodies overnight (4 °C), sections were incubated in polymer-HRP conjugated secondary antibody (Dako) for 2 h at room temperature, followed by DAB (Thermo-fisher) or Opal-520, 570, 650, 690 TSA dye (Akoya biosciences) development. DAB-stained section images were captured by the Nikon Ni-U Light Microscope and analyzed using Image J analysis software; Opal TSA-stained sections were captured on the Mantra quantitative pathology workstation (Akoya Biosciences) and analyzed by inForm image analysis software 2.6 (Akoya Biosciences) as per our previous studies^18,51^.

### Tissue array study

Archival formalin-fixed paraffin-embedded (FFPE) tissue specimens were retrieved from 72 lung adenocarcinoma (LUAD) patients with complete patient demographic data (included male and female, aged between 37 and 85) who underwent surgery at the Prince of Wales Hospital, Hong Kong SAR. Data are presented as frequency (%) with analysis using the Spearman correlation coefficient. iNOS^+^CD16b^+^ and CD206^+^CD16b^+^ were defined as N1 and N2 phenotypes, respectively^36^. Kaplan-Meier analysis and log-rank test were used to evaluate the difference in survival of patients stratified into different groups based on the frequency of N1 TANs as a proportion of total TANs. The determination of high and low N1 TAN cut-offs was based on median values. Clinical information and the relative marker quantification of tissue arrays are listed in Supplementary Data 1.

### Immunofluorescence

Optimal cutting temperature compound embedded frozen tumor tissues sections (5 µm) were incubated with CD206, iNOS, Ly6G, and p-Smad3 antibodies overnight at 4 °C, followed by incubation with Alexa Fluor 546-conjugated or Alexa Four 488-conjugated secondary antibodies (1:1,000; Invitrogen) in staining buffer (eBioscience) for 2 h at room temperature. After washing with PBS, the sections were stained with FITC-conjugated Ly6G or PE-conjugated CD16b overnight at 4 °C. Nuclei were stained with Hoechst 33342 (Invitrogen) for 5 min in PBS, then mounted with PermaFluor medium (Thermo Fisher Scientific)^52^. Images and z-stack scanning were acquired by Zeiss Axio Observer.Z1 and LSM 880 fluorescence microscopes, respectively, and analyzed with ZEN lite image analysis software 2.4.

### Flow cytometric analysis

Tissues isolated from tumor-bearing mice were mechanistically dissociated, digested by Liberase™ TM (Roche), filtered through 40-μm nylon mesh, and fixed with IC Fixation Buffer (eBioscience) according to manufacturer’s protocol to prepare single-cell suspensions. Neutrophils were stained with Ly6G, CD11b, iNOS and CD206 antibodies overnight at 4 °C. Flow cytometric data were acquired on LSRFortessa (Becton Dickinson) and analyzed with the Cytobank platform (cytobank.org) for quantitative analysis.

### 10x scRNA-seq and transcriptomic analysis

Lung tumors from LLC inoculated mice (Both Smad3-WT and KO mice, 8 tumors/group) were dissected, digested into a single-cell population, and then CD11b^+^Ly6G^+^ cells isolated by FACS. Sorted cells were encapsulated and library construction was performed with the Chromium controller with a 5′ expression kit (10x Genomics). For each group, eight tumor samples were mixed to generate single-cell droplets. Libraries were sequenced on the Illumina NovaSeq 6000 platform (PE151bp, 660 M raw read). Data processing, analysis, and visualization were performed in R studio (v1.3.1073) using Seurat (v4.1.1). For quality control, cells with at least 200 detected genes were selected for analysis, with the percentage of mitochondrial-derived reads set at less than 10%, and the total number of molecules within each cell was filtered by 500 <nCount RNA <20,000. A total of 4116 cells from Smad3-WT TANs and 2514 cells from Smad3-KO TANs were sorted with a median of 1311 unique molecular identifiers (UMIs) and 603 genes per cell. Raw Fastq data were processed using the Cell Ranger v3.0.2 pipeline to generate an expression matrix and converted into cloupe format. Dimensionality reduction was performed through Cell Ranger v3.0.2 pipeline, with 100 principal components used to compute a t-Distributed stochastic neighbor embedding (t-SNE) of cells with van der Maaten method and Louvain Modularity Optimization^53^. Neutrophil-specific markers (S100a9, S100a8, Csf3r, and Il1rn) were applied to filter Smad3-WT and Smad3-KO TANs datasets, resulting in 3293 Smad3-WT TANs and 1323 Smad3-KO TANs retained for analysis^22^. Smad3-WT and -KO TANs were subsequently merged into a single dataset with eight informative clusters (P1–P8). The clusters and differentially expressed genes (DEGs) were mined using Loupe Cell Browser software 6.0^22,54^. The scRNA-seq raw data have been deposited in the GEO database (GSE186530). Upregulated DEGs of Smad3-WT (P1) and Smad3-KO (P8) specific clusters of TANs were extracted and submitted to the Database for Annotation, Visualization, and Integrated Discovery bioinformatics resources (DAVID v6.8) for Gene Ontology biological process enrichment analysis. All selections were filtered by Fisher’s exact *P* value of <0.05.

We used the MetaCell package to select feature genes related to neutrophil development^26^, construct cell clusters along transcriptional gradients (termed metacells), and visualize and rebuild a k-NN graph (Fig. 4D) based on t-SNE data in Fig. 3A^25^. Cells that contained less than 500 UMIs or had a mitochondrial transcript fraction of >0.2 were removed. Next, variable genes across the dataset were identified with a normalized variance/mean threshold at 0.1 and a downsampled coverage threshold at 80, yielding genes were subsequently used as anchors to search for gene–gene correlations across the dataset, and genes with correlations of >0.1 were included.

The public human non-small cell lung cancer (NSCLC) scRNA-seq dataset of tumor cells from seven donors with a complete description of sample collection, library preparation, and sequencing configuration can be accessed on 10x Genomics website via the following URL: https://www.10xgenomics.com/resources/datasets/40-k-mixture-of-nsclc-dt-cs-from-7-donors-3-ht-v-3-1-3-1-high-6-1-0. Clustering of Uniform Manifold Approximation and Projection (UMAP) was performed by Cell Ranger pipeline with default parameters^55^. Neutrophil were extracted from the NSCLC dataset by Loupe Cell Browser software 6.0 following protocol from the 10x Genomics website via the following URL: https://support.10xgenomics.com/single-cell-gene-expression/software/pipelines/latest/tutorials/neutrophils. SMAD3 + and SMAD3- TANs were re-clustered through dimensionality reduction with UMAP and expression of neutrophils marker genes (CSF3R, FPR1, NAMPT, MNDA, and FCGR3B) (Supplementary Fig. 7).

### RNA velocity and developmental trajectory analysis

RNA velocity analysis was performed by scVelo to infer the future states of individual cells using the spliced and unspliced information as described in previous studies^18,56^. The aligned bam file generated by Cell Ranger was recounted with the Velocyto counting pipeline velocyto.py in python. The complete code and notebooks of Velocyto and scVelo utilized is available at http://velocyto.org. and https://scvelo.readthedocs.io. The sample-wise counts of unspliced and spliced reads in loom format were loaded to scVelo. Genes with less than 20 spliced and unspliced counts in a cell were filtered, and the counts were normalized using normalize_per_cell. A total of 2000 high variability genes were identified and retained by filter_genes_dispersion, following which the counts were log-transformed using log1p. The first- and second-order moments for each cell across its nearest neighbors were calculated using scvelo.pp.moments. Subsequently, the velocities were estimated using the scvelo.tl.velocity and the velocity graph constructed using scvelo.tl.velocity_graph function. Velocities were visualized on top of the diffusion UMAP space. The confidence values of the RNA velocity were computed with scVelo.tl.velocity_confidence function. Developmental trajectories of Smad3-WT and Smad3-KO TANs were reconstructed using the Monocle package; N1 (Tnf^+^) and N2 (Ccl2^+^) phenotypes were annotated according to *Tnf* and *Ccl2* expression, respectively.

### Untouched bone marrow-derived neutrophils (BMDN)

Bone marrow from the tibia, femur, and ilium was harvested from LLC tumor-bearing mice, filtered using 40-μm nylon mesh and decontaminated from erythrocytes to prepare single-cell suspension as described in our previous study^17^. BMDNs were isolated using the Untouched Neutrophil Isolation Kit (130-097-658, Miltenyi Biotec) according to the manufacturer’s instruction and maintained with Iscove’s Modified Dulbecco’s Medium (Gibco) supplemented with 1% horse serum (Rockland) and 10 ng/ml granulocyte-macrophage colony-stimulating factor (GM-CSF) (Invitrogen).

### Untouched peripheral blood-derived neutrophils (PBDN)

PBDNs were isolated from healthy human buffy coat samples using the Whole Blood Neutrophil Isolation Kit (130-104-434, Miltenyi Biotec) according to manufacturer’s instructions and kept in RPMI 1640 (Gibco) supplemented with 1% horse serum (Rockland) and 10 ng/ml GM-CSF (Invitrogen).

### Anti-Ly6G treatment

Anti-Ly6G antibody (clone 1A8, #BE0075-1)^28^ (50 µg/mouse) was given by intraperitoneal injection to Smad3-WT and Smad3-KO mice 3 days before subcutaneous inoculation with LLC cells, and then given every day until animals were killed. The control group received an IgG isotype control antibody. Tumor size was monitored with a Vernier caliper every 2 days along the treatment course.

### Adoptive transfer study

BMDN isolated from tumor-bearing Smad3-WT and Smad3-KO donors were adoptively transferred into LLC-bearing C57BL/6 J mice. In brief, untouched Smad3-WT and Smad3-KO BMDNs (3 × 10^6^ cells/mouse) were given to recipient mice via tail-vein injection on day 15 and day 20 after LLC tumor establishment, and mice were killed on day 25. This timing was chosen as neutrophil infiltration in the inflammatory site is diminished after 5 days^12^. PBDNs transfected with a scrambled nonsense control siRNA (NC-PBDN) or with SMAD3 siRNAs (S3KD-PBDN) were adoptively transferred into A549-bearing NSG mice via tail-vein infusion on days 15 and 20 after tumor establishment^17^. Tumor volume was measured with a Vernier caliper and calculated using the equation: Volume (mm^3^) = 0.5 (long × square of short diameter).

### Adoptive transfer of DiI/CFSE-labeled BMDNs

Untouched BMDNs were isolated from Smad3-WT and Smad3-KO mice and then labeled with CellTrace™ CFSE Solution (green, Invitrogen) or Vybrant™ DiI Cell-Labeling Solution (red, Invitrogen), respectively, following the manufacturer’s protocol. A 1:1 mixture of CFSE-labeled Smad3-WT-BMDN and DiI-labeled Smad3-KO-BMDN was injected (3 × 10^6^ cells/mouse) into C57BL/6 J mice on Day 15 after inoculation with LLC cells. Mice were killed on Day 16 and tumors were collected.

### Cytotoxicity assay

Untouched Smad3-WT and Smad3-KO BMDNs were co-cultured with LLC cells at ratios of 1:5, 1:10, and 1:20 (LLC: BMDN) for 6 h. In addition, NC- and siSMAD3- treated PBDNs were co-cultured with A549 cells at ratios of 1:2, 1:5, and 1:10 (A549: PBDN) for 6 h. Supernatants were then collected by centrifugation and analysed for LDH release using the CytoTox 96® Non-Radioactive Cytotoxicity Assay (Promega) according to the manufacturer’s instructions.

### Phagocytosis assay (Dil-LLC uptake assay)

LLC cells (1 × 10^7^) were incubated with 50 μL of Vybrant™ DiI Cell-Labeling Solution (Invitrogen, 5 μg/μL in ethanol) for 20 min at 37 °C in the dark. Unbound dye was removed by PBS washing and centrifugation at 500×*g* for 5 min. Untouched Smad3-WT and Smad3-KO BMDNs were co-cultured with Dil-LLC cells at ratios of 1:5 and 1:10 (LLC: BMDN) for 6 h. Fluorescent intensity (excitation 583 nm and emission 475 nm) was detected by a spectrophotometer (Molecular Devices)^13^. Unstained LLC cells and BMDNs were included as negative controls.

For phagocytosis assay-related flow cytometric analysis^57^, untouched Smad3-WT and Smad3-KO BMDNs were co-cultured with Dil-LLC cells at ratios of 1:5 (LLC: BMDN) for 6 h. Flow cytometric data were acquired on LSRFortessa (Becton Dickinson) and analyzed with the Cytobank platform (cytobank.org) for quantitative analysis.

### ChIP-sequencing

Bone marrow-derived neutrophil cells extracted from Smad3-WT mice (pooled from four mice /group) were treated with 10% LLC-CM or normal media (control) for 2 h, then processed using the SimpleChIP Enzymatic Chromatin IP Kit (Cell Signaling Technology) according to the manufacturer’s instruction^58^. Antibodies against Smad3 and IgG isotype control (Cell Signaling Technology) were used for immunoprecipitation. Smad3 immuno-enriched DNA (input and Smad3-IP) were sequenced using the Illumina HiSeq platform (PE150bp, 40 M raw read), mapped against GRCm38 (mm10) *Mus musculus* genome using Bowtie2, and peaks identified using model-based analysis for ChIP-Seq (MACS) with default parameters. Normalized Smad3 binding regions were visualized by EaSeq^59^, and annotated by the Genomic Regions Enrichment of Annotations Tool (GREAT) for DAVID-GO enrichment analysis. Motif analysis was performed in Trawler (trawler.erc.monash.edu.au) with default parameters.

### Real-time PCR analysis

Total cellular RNA was extracted using the Trizol reagent (Molecular Research Center), reverse transcribed, and quantified by real-time PCR using SYBR Green Supermix (Life Technologies, Carlsbad, CA, USA) with primers as previously described in refs. ^17,60^. The primers used are listed in Supplementary Table 4. The relative expression of target genes was normalized against the internal control GAPDH and calculated by the 2^−ΔΔCt^ method.

### siRNA knockdown

Human PBDNs were harvested and transfected with 100 nM of a scrambled nonsense control (siN05815122147, RIBOBIO) or siRNA against SMAD3 (5′-AAGAGAUUCGAAUGACGGUAA-3′). The knockdown efficiency was quantified by real-time PCR with primers for human SMAD3 and GAPDH (see Supplementary Table 4).

### SIS3 treatment

Tumor-bearing Smad3-WT mice were randomly divided into four groups on Day 7 after inoculation with LLC cells. Mice were administered 2.5, 5, or 10 μg/g SIS3 (S7959, Selleckchem) by daily intraperitoneal injection from day 7 until being killed on day 16 for tumor harvest. The control group received vehicle control (0.05% dimethyl sulfoxide)^17^. Tumor size was monitored with a Vernier caliper. For in vitro experiments, BMDN cells were pre-treated with or without SIS3 (2 μM/mL) for 2 h and then stimulated with LLC-CM for 24 h.

### Statistical analysis

Two-tailed student’s *t*-test and analysis of variance with Bonferroni’s correction were used for statistical analysis of the differences in mRNA expression levels, tumor weight and growth, neutrophil infiltration, viability, cytotoxicity, and phagocytosis, flow cytometric analysis, IHC and IF quantification using the Prism program (Prism 5.0, 9.0 GraphPad Software, San Diego, CA). Data are presented as mean ± SEM. Spearman correlation analysis and log-rank test were used to analyze the NSCLC cohort data. *P* value < 0.05 was considered statistically significant.

### Reporting summary

Further information on research design is available in the Nature Portfolio Reporting Summary linked to this article.

## Supplementary information

Supplementary Information

Description of Additional Supplementary Files

Supplementary Data 1

Supplementary Data 2

Supplementary Data 3

Reporting Summary

## Data Availability

The raw single-cell RNA-seq and ChIP-seq datasets are available in the Gene Expression Omnibus and ArrayExpress database under the accession number GSE186530 and E-MTAB-12740, respectively. The human NSCLC publicly available data used in this study are available in the 10X genomics database [https://www.10xgenomics.com/resources/datasets/40-k-mixture-of-nsclc-dt-cs-from-7-donors-3-ht-v-3-1-3-1-high-6-1-0]. The remaining data are available within the Article, Supplementary Information or Source Data file. Source data are provided with this paper.

## Source data

Source Data

## References

[1] Kong X (2017). Cancer drug addiction is relayed by an ERK2-dependent phenotype switch. Nature.

[2] Bray F (2018). Global cancer statistics 2018: GLOBOCAN estimates of incidence and mortality worldwide for 36 cancers in 185 countries. CA Cancer J. Clin..

[3] Boyero L (2020). Primary and acquired resistance to immunotherapy in lung cancer: unveiling the mechanisms underlying of immune checkpoint blockade therapy. Cancers.

[4] Yarchoan M, Hopkins A, Jaffee EM (2017). Tumor mutational burden and response rate to PD-1 inhibition. N. Engl. J. Med..

[5] Schreiber RD, Old LJ, Smyth MJ (2011). Cancer immunoediting: integrating immunity’s roles in cancer suppression and promotion. Science.

[6] Banat GA (2015). Immune and inflammatory cell composition of human lung cancer stroma. PLoS ONE.

[7] Coffelt SB, Wellenstein MD, de Visser KE (2016). Neutrophils in cancer: neutral no more. Nat. Rev. Cancer.

[8] Jaillon S (2020). Neutrophil diversity and plasticity in tumour progression and therapy. Nat. Rev. Cancer.

[9] Dahmani A, Delisle JS (2018). TGF-beta in T cell biology: implications for cancer immunotherapy. Cancers.

[10] Suzuki E (2007). A novel small-molecule inhibitor of transforming growth factor beta type I receptor kinase (SM16) inhibits murine mesothelioma tumor growth in vivo and prevents tumor recurrence after surgical resection. Cancer Res..

[11] Fridlender ZG (2009). Polarization of tumor-associated neutrophil phenotype by TGF-beta: "N1" versus "N2" TAN. Cancer Cell.

[12] Kim MH (2008). Dynamics of neutrophil infiltration during cutaneous wound healing and infection using fluorescence imaging. J. Invest. Dermatol..

[13] Phang SW, Ooi BK, Ahemad N, Yap WH (2020). Maslinic acid suppresses macrophage foam cells formation: regulation of monocyte recruitment and macrophage lipids homeostasis. Vasc. Pharm..

[14] Chung JY (2021). TGF-beta signaling: from tissue fibrosis to tumor microenvironment. Int. J. Mol. Sci..

[15] Chan MK (2022). TGF-beta signaling networks in the tumor microenvironment. Cancer Lett..

[16] Marwitz S (2021). Phosphorylation of SMAD3 in immune cells predicts survival of patients with early stage non-small cell lung cancer. J. Immunother. Cancer.

[17] Tang PM (2017). Smad3 promotes cancer progression by inhibiting E4BP4-mediated NK cell development. Nat. Commun..

[18] Tang PC (2022). Smad3 promotes cancer-associated fibroblasts generation via Macrophage-Myofibroblast Transition. Adv. Sci. (Weinh.).

[19] Golay J (2013). Glycoengineered CD20 antibody obinutuzumab activates neutrophils and mediates phagocytosis through CD16B more efficiently than rituximab. Blood.

[20] Tsuboi N, Asano K, Lauterbach M, Mayadas TN (2008). Human neutrophil Fcgamma receptors initiate and play specialized nonredundant roles in antibody-mediated inflammatory diseases. Immunity.

[21] Jonsson F (2011). Mouse and human neutrophils induce anaphylaxis. J. Clin. Invest.

[22] Zilionis R (2019). Single-cell transcriptomics of human and mouse lung cancers reveals conserved myeloid populations across individuals and species. Immunity.

[23] Shaul ME (2016). Tumor-associated neutrophils display a distinct N1 profile following TGFbeta modulation: a transcriptomics analysis of pro- vs. antitumor TANs. Oncoimmunology.

[24] La Manno G (2018). RNA velocity of single cells. Nature.

[25] Baran Y (2019). MetaCell: analysis of single-cell RNA-seq data using K-nn graph partitions. Genome Biol..

[26] Grieshaber-Bouyer R (2021). The neutrotime transcriptional signature defines a single continuum of neutrophils across biological compartments. Nat. Commun..

[27] Evrard M (2018). Developmental analysis of bone marrow neutrophils reveals populations specialized in expansion, trafficking, and effector functions. Immunity.

[28] Boivin G (2020). Durable and controlled depletion of neutrophils in mice. Nat. Commun..

[29] Zhang YY (2019). LRNA9884, a novel Smad3-dependent long noncoding RNA, promotes diabetic kidney injury in db/db mice via enhancing MCP-1-dependent renal inflammation. Diabetes.

[30] Chapouly C (2020). Desert hedgehog-driven endothelium integrity is enhanced by Gas1 (growth arrest-specific 1) but negatively regulated by Cdon (cell adhesion molecule-related/downregulated by oncogenes). Arterioscler Thromb. Vasc. Biol..

[31] Mathew E (2014). Dosage-dependent regulation of pancreatic cancer growth and angiogenesis by hedgehog signaling. Cell Rep..

[32] Robichaud N (2018). Translational control in the tumor microenvironment promotes lung metastasis: Phosphorylation of eIF4E in neutrophils. Proc. Natl Acad. Sci. USA.

[33] Wang L, Sparks-Wallace A, Casteel JL, Howell MEA, Ning S (2021). Algorithm-based meta-analysis reveals the mechanistic interaction of the tumor suppressor LIMD1 with non-small-cell lung carcinoma. Front. Oncol..

[34] Kuwabara WMT, Andrade-Silva J, Pereira JNB, Scialfa JH, Cipolla-Neto J (2019). Neutrophil activation causes tumor regression in Walker 256 tumor-bearing rats. Sci. Rep..

[35] Batlle E, Massague J (2019). Transforming growth factor-beta signaling in immunity and cancer. Immunity.

[36] Tyagi A (2021). Nicotine promotes breast cancer metastasis by stimulating N2 neutrophils and generating pre-metastatic niche in lung. Nat. Commun..

[37] Mishalian I (2013). Tumor-associated neutrophils (TAN) develop pro-tumorigenic properties during tumor progression. Cancer Immunol. Immunother..

[38] Tufegdzic Vidakovic A (2015). Context-specific effects of TGF-beta/SMAD3 in cancer are modulated by the epigenome. Cell Rep..

[39] Burdon PC, Martin C, Rankin SM (2005). The CXC chemokine MIP-2 stimulates neutrophil mobilization from the rat bone marrow in a CD49d-dependent manner. Blood.

[40] Xu H (2022). Lentinan enhances the antitumor effects of Delta-like 1 via neutrophils. BMC Cancer.

[41] Tabaries S (2015). Granulocytic immune infiltrates are essential for the efficient formation of breast cancer liver metastases. Breast Cancer Res..

[42] Zhong J, Li Q, Luo H, Holmdahl R (2021). Neutrophil-derived reactive oxygen species promote tumor colonization. Commun. Biol..

[43] Zhao L (2018). Pharmacological activation of estrogen receptor beta augments innate immunity to suppress cancer metastasis. Proc. Natl Acad. Sci. USA.

[44] Nakayama KH, Hou L, Huang NF (2014). Role of extracellular matrix signaling cues in modulating cell fate commitment for cardiovascular tissue engineering. Adv. Health. Mater..

[45] Anthoni M, Fyhrquist-Vanni N, Wolff H, Alenius H, Lauerma A (2008). Transforming growth factor-beta/Smad3 signalling regulates inflammatory responses in a murine model of contact hypersensitivity. Br. J. Dermatol.

[46] Flanders KC (2008). Absence of Smad3 induces neutrophil migration after cutaneous irradiation: possible contribution to subsequent radioprotection. Am. J. Pathol..

[47] Chu D, Dong X, Shi X, Zhang C, Wang Z (2018). Neutrophil-based drug delivery systems. Adv. Mater..

[48] Xue J (2017). Neutrophil-mediated anticancer drug delivery for suppression of postoperative malignant glioma recurrence. Nat. Nanotechnol..

[49] Wu N (2020). Discovery of a novel selective water-soluble SMAD3 inhibitor as an antitumor agent. Bioorg. Med. Chem. Lett..

[50] Yang X (1999). Targeted disruption of SMAD3 results in impaired mucosal immunity and diminished T cell responsiveness to TGF-beta. EMBO J..

[51] Li C (2020). The Mincle/Syk/NF-kappaB signaling circuit is essential for maintaining the protumoral activities of tumor-associated macrophages. Cancer Immunol. Res..

[52] Xue VW (2021). USMB-shMincle: a virus-free gene therapy for blocking M1/M2 polarization of tumor-associated macrophages. Mol. Ther. Oncolytics.

[53] Maaten LVD (2014). Accelerating t-SNE using tree-based algorithms. J. Mach. Learn. Res..

[54] Zhang Y (2020). Long non-coding RNA LRNA9884 promotes acute Kidney injury via regulating NF-kB-mediated transcriptional activation of MIF. Front. Physiol..

[55] Becht, E. et al. Dimensionality reduction for visualizing single-cell data using UMAP. *Nat. Biotechnol*. **37**, 38–44 (2018). 10.1038/nbt.4314 30531897

[56] Tang PC (2022). Single-cell RNA sequencing uncovers a neuron-like macrophage subset associated with cancer pain. Sci. Adv..

[57] Wang Y (2022). Extracellular HMGB1 impairs macrophage-mediated efferocytosis by suppressing the Rab43-controlled cell surface transport of CD91. Front. Immunol..

[58] Tang PM (2018). The proto-oncogene tyrosine protein kinase Src is essential for macrophage-myofibroblast transition during renal scarring. Kidney Int..

[59] Lerdrup M, Johansen JV, Agrawal-Singh S, Hansen K (2016). An interactive environment for agile analysis and visualization of ChIP-sequencing data. Nat. Struct. Mol. Biol..

[60] Tang, P. M. et al. DPP4/CD32b/NF-kappaB circuit: a novel druggable target for inhibiting CRP-driven diabetic nephropathy. *Mol. Ther.* **29**, 365–375 (2020). 10.1016/j.ymthe.2020.08.017 PMC7790911 32956626

