# Supplementary material for: Smad3 is essential for polarization of tumor-associated neutrophils in non-small cell lung carcinoma
Source: Nat Commun. 2023 Mar 31;14:1794. doi: 10.1038/s41467-023-37515-8 (PMC10066366; doi:10.1038/s41467-023-37515-8)
Supplement: Supplementary file 1 — Supplementary Information [file 41467_2023_37515_MOESM1_ESM.pdf]

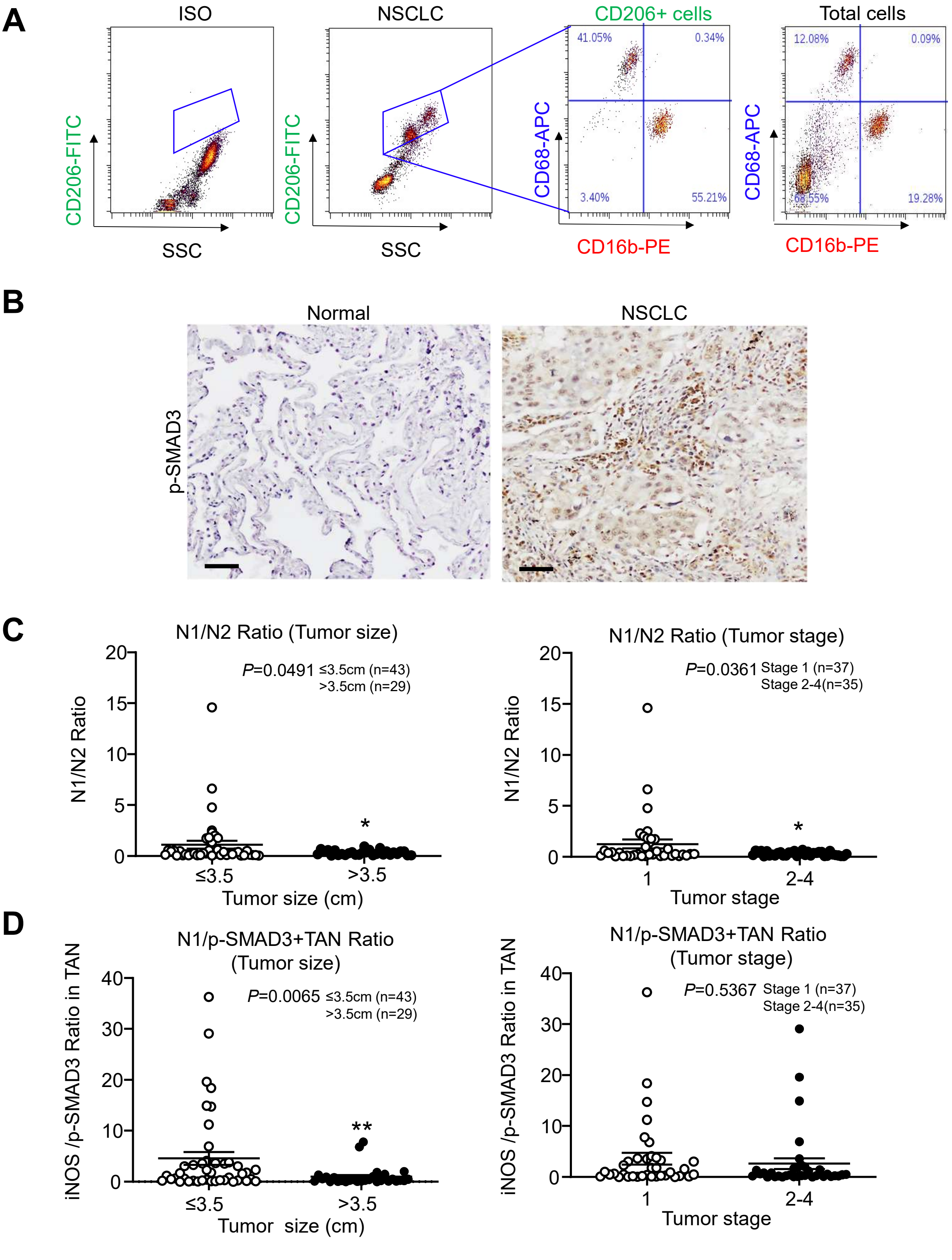

Supplementary Fig 1. Identification of N1 and N2 populations and their association with progression in human NSCLC. A. Flow cytometry analysis of a fresh NSCLC tumor. Analysis shows that CD206+ cells contain distinct populations of CD68+ monocyte/macrophages and CD16b+ neutrophils. **B.** Immunoperoxidase staining (brown, with haematoxylin nuclear counterstain) for phosphorylation (activation) of SMAD3 in sections of NSCLC and adjacent normal lung control tissue. While few p-SMAD3+ cells are evident in normal lung, many p-SMAD3+ cells are evident within the tumor. **C.** Graphs showing that the N1/N2 ratio of tumor-associated neutrophils (TAN) is significantly lower in tumor of larger size and more advanced stage.(two tailed Welch's t test) **D.** Graphs showing a significant reduction in the N1/p-SMAD3 ratio within TANs with larger tumor size, but not with tumor stage.(two tailed Welch's t test) (**A-B**) Data represents of 4 patients/group (**B**) three independent experiments show similar results. (**C-D**) Data represents mean ± SEM from 72 subjects. Scale bars, 50 µm. Source data are provided as a Source Data file

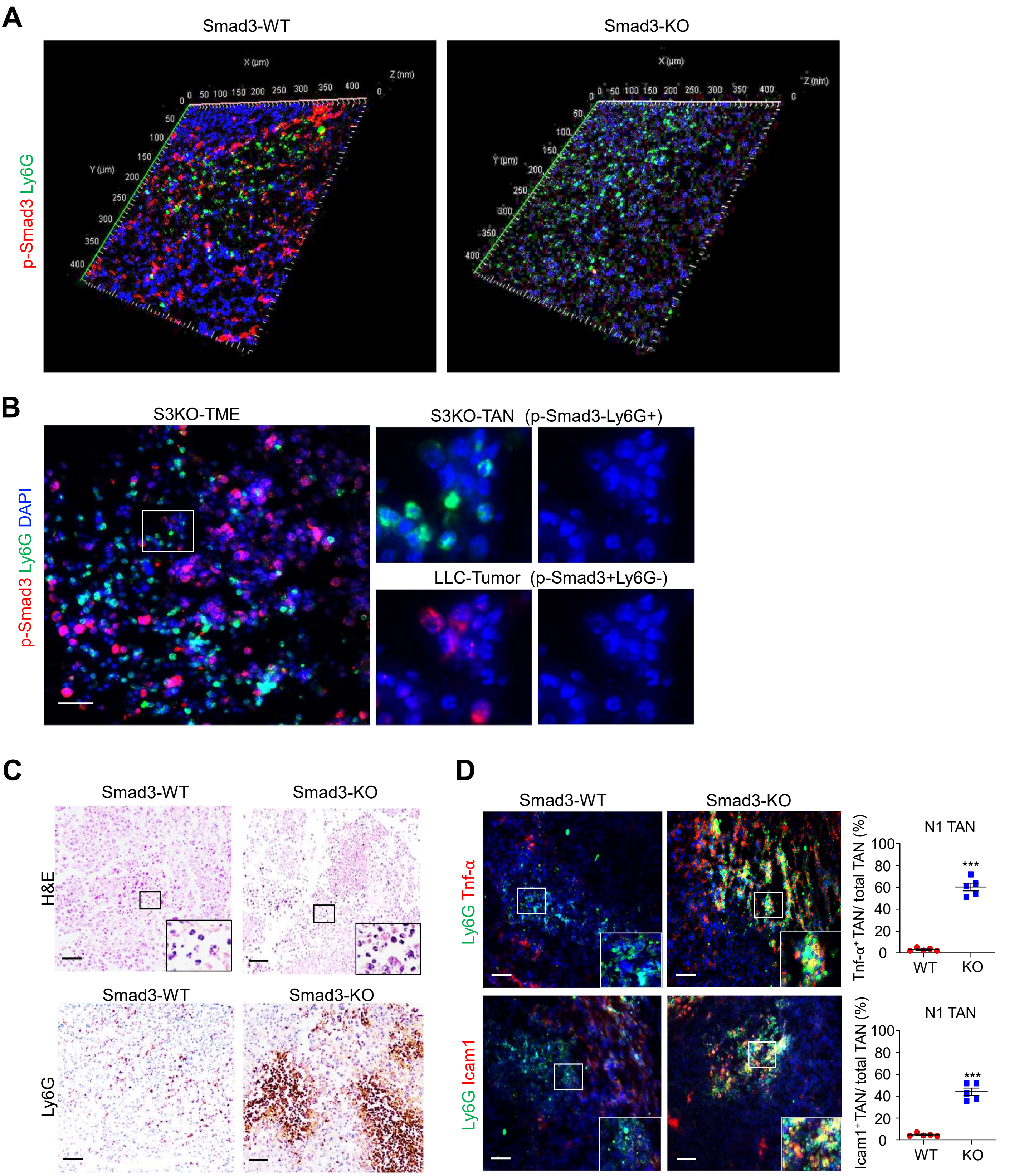

Supplementary Fig 2. Increase of tumor-infiltrating TANs in the LLC-tumor with Smad3-KO TME *in vivo*.

A. 3-dimenisional confocal images show the distinct infiltration patterns of Smad3-WT and -KO TAN in the LLC-tumours. **C.** H&E and immunohistochemistry staining also increased neutrophil infiltration in terms of neutrophil morphology and Ly6G expression in the stroma of LLC tumor from Smad3-KO mice. **B.** Immunofluorescence staining show no p-Smad3 expression in S3KO-TAN. **D.** N1 phenotype (TNF-α+Ly6G/ Icam1+Ly6G) is increased in the TME of LLC-bearing Smad3-KO mice compared to the Smad3-WT mice. (****P*<0.001 vs. Control, ###*P*<0.001 vs. WT-BMDN, one-way ANOVA). Scale bars, 50 µm. (**C-D**) Two independent experiments show similar results. **(A-D)** Data represents of 5 mice/group. (**D**) Data represents mean ± SEM of 5 mice/group. Exact P values of Smad3-WT vs. Smad3-KO are **2D**. *P*=0.0001(Tnf-α+TAN), *P*= 0.0005(Icam1+ TAN). Source data are provided as a Source Data file

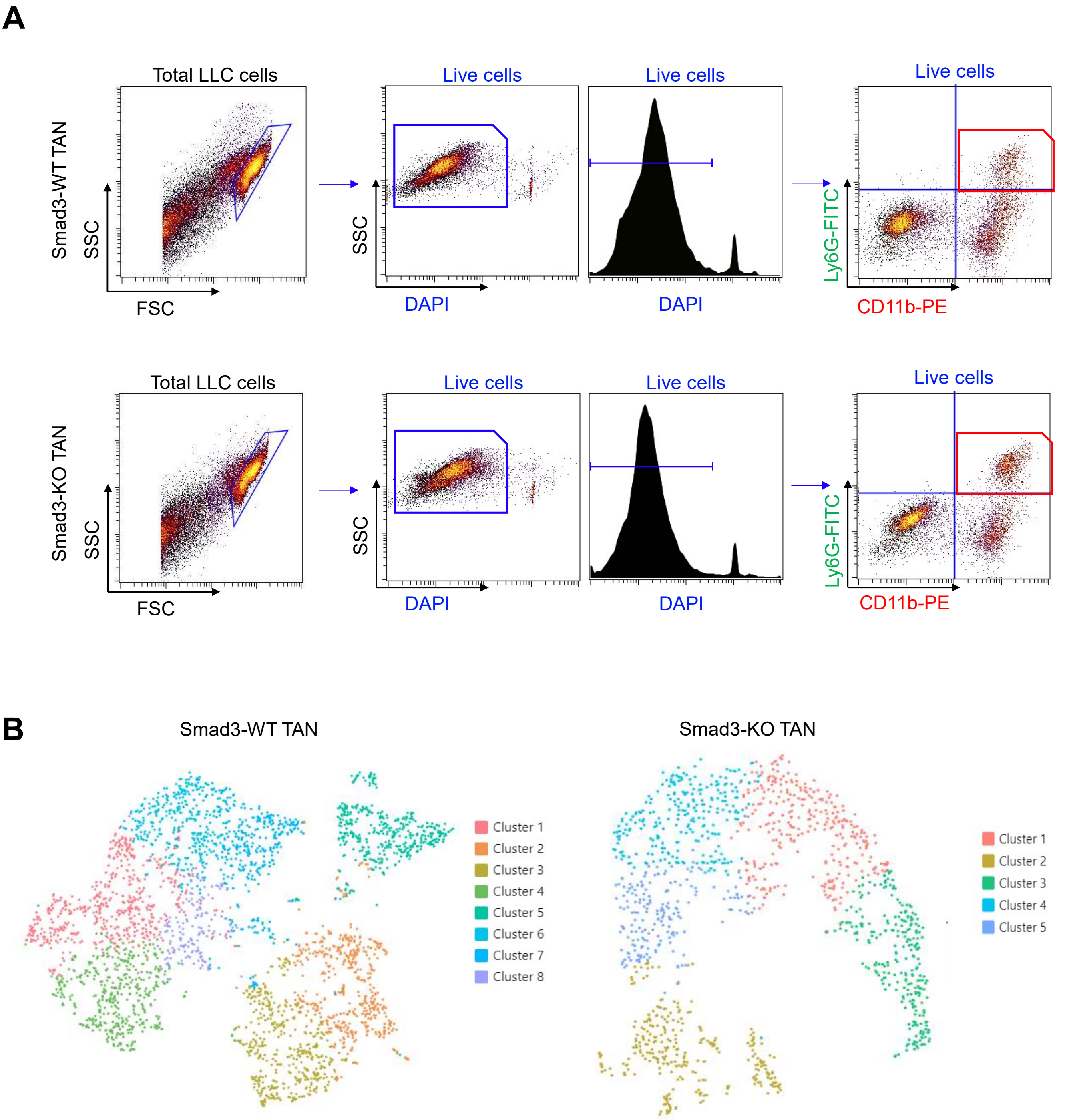

Supplementary Fig 3. Gating strategy and individual plot of the TAN-specific 10X scRNA-seq.
A. The live cells of LLC-tumor tissue were identified with DAPI exclusion, then the viable CD11b+ Ly6G+ Smad3-WT and –KO TANs were collected by FACS and submitted for 10X scRNA-seq analysis. **B.** Individual t-SNE plot of the Smad3-WT and –KO TAN 10x scRNA-seq datasets.

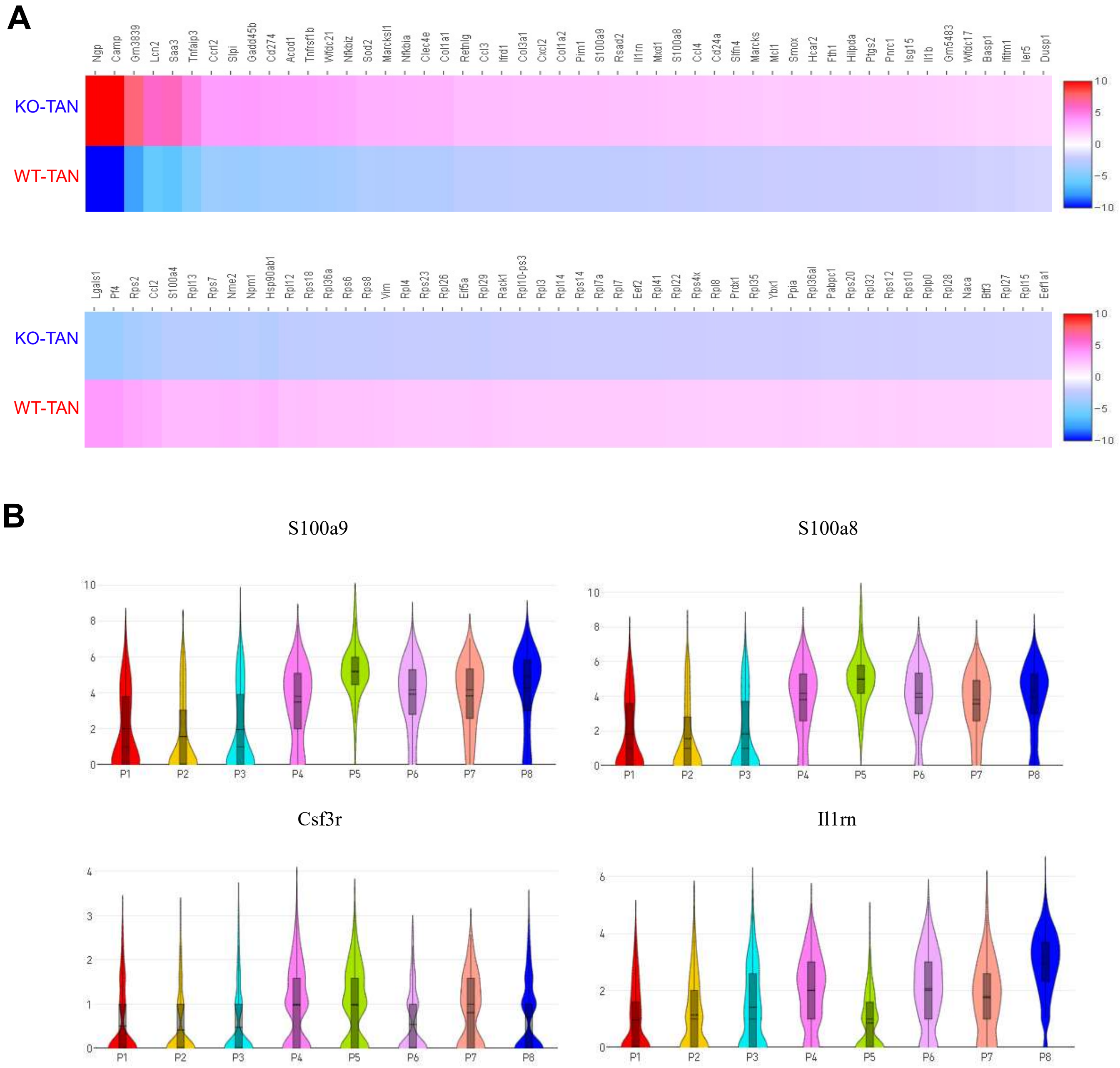

Supplementary Fig 4. Expression levels of neutrophil specific markers in the cleaned TAN-specific scRNA-seq.
A. Heatmaps of top 50 up-regulated and downregulated genes in the TANs of Smad3-WT and -KO mice. **B.** Expression level (Log2 fold change) of reported neutrophil specific markers in all clusters of the TAN scRNA-seq in Figure 3D (n=447 cells for P1, n=579 cells for P2, n=653 cells for P3, n=589 cells for P4, n=671 cells for P5, n=685 cells for P6, n=174 cells for P7, n=818 cells for P8). (B) Each box represents the IQR and median of the UMI counts per cell of each cell cluster, whiskers indicate 1.5 times IQR, endpoints depict minimum and maximum values.

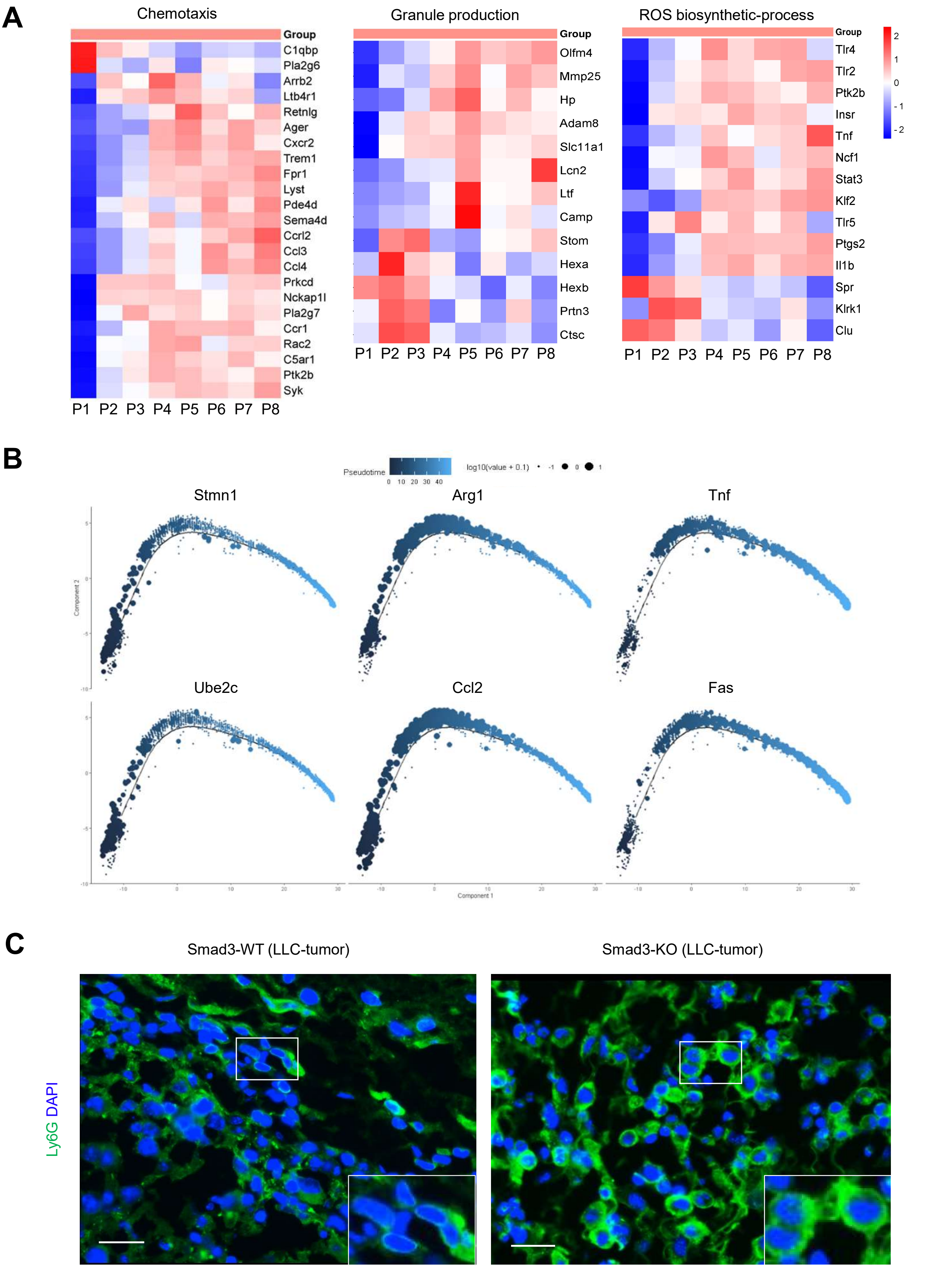

Supplementary Fig 5. Characteristics of each cell cluster (P1-P8) in the TAN-specific scRNA-seq dataset. A. Chemotaxis, Granule production and ROS biosynthetic-process-related genes expression in each TAN cluster, shows P1-P3 were immature neutrophil, P4-P8 are mature neutrophil. **B.** Neutrophil development gene expression with N1/N2 marker along pseudo-time shows Smad3 deficiency mature the TAN development and promote the TANs into N1 phenotype. **C.** Immunofluorescence staining show different nucleus size of Smad3-WT and -KO TANs in the LLC-tumor in vivo. Scale bars, 50 μm. (**C**) Data represents of 5 mice/group. Two independent experiments show similar results.

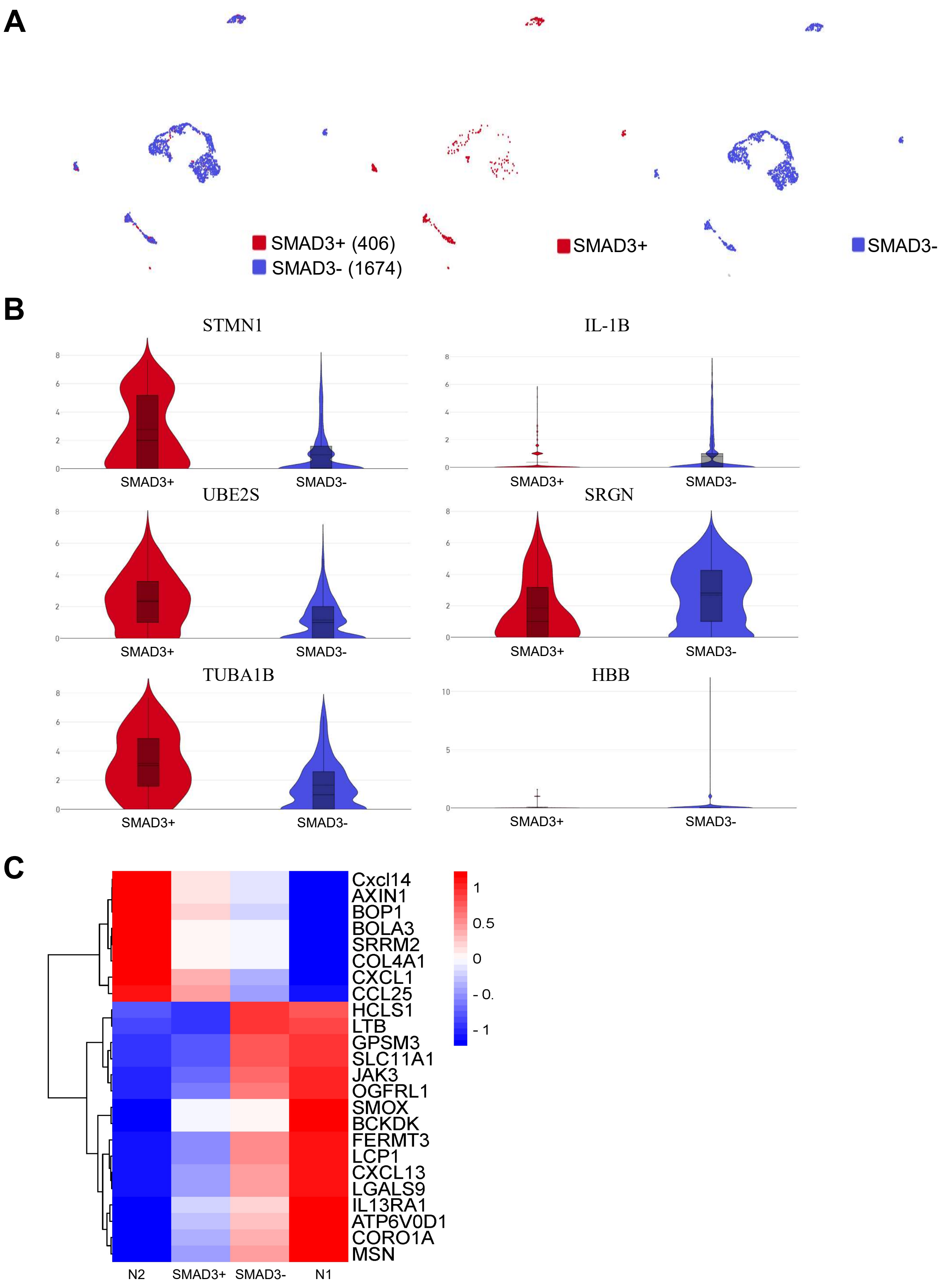

Supplementary Fig 6. Expression levels of neutrophil markers in the NSCLC scRNA-seq public dataset.
A. UMAP of the human TAN cluster (SMAD3+ and SMAD3-) re-cluster from 10x NSCLC public dataset with human neutrophil marker (CSF3R, FPR1, NAMPT, MNDA and FCGR3B). **B.** Expression level of neutrophil development gene expression (immature marker: STMN1, UBE2S and TUBA1B, mature marker: IL-1B, SRGN and HBB) in human TAN cluster. (n=406 cells for SMAD3+ TANs, n=1674 cells for SMAD3- TANs) **C.** Heatmap shows SMAD3+ and SMAD3- human TAN clusters share similar transcriptome signatures with N2 and N1 TANs, respectively. (B) Each box represents the IQR and median of the UMI counts per cell of each cell cluster, whiskers indicate 1.5 times IQR, endpoints depict minimum and maximum values.

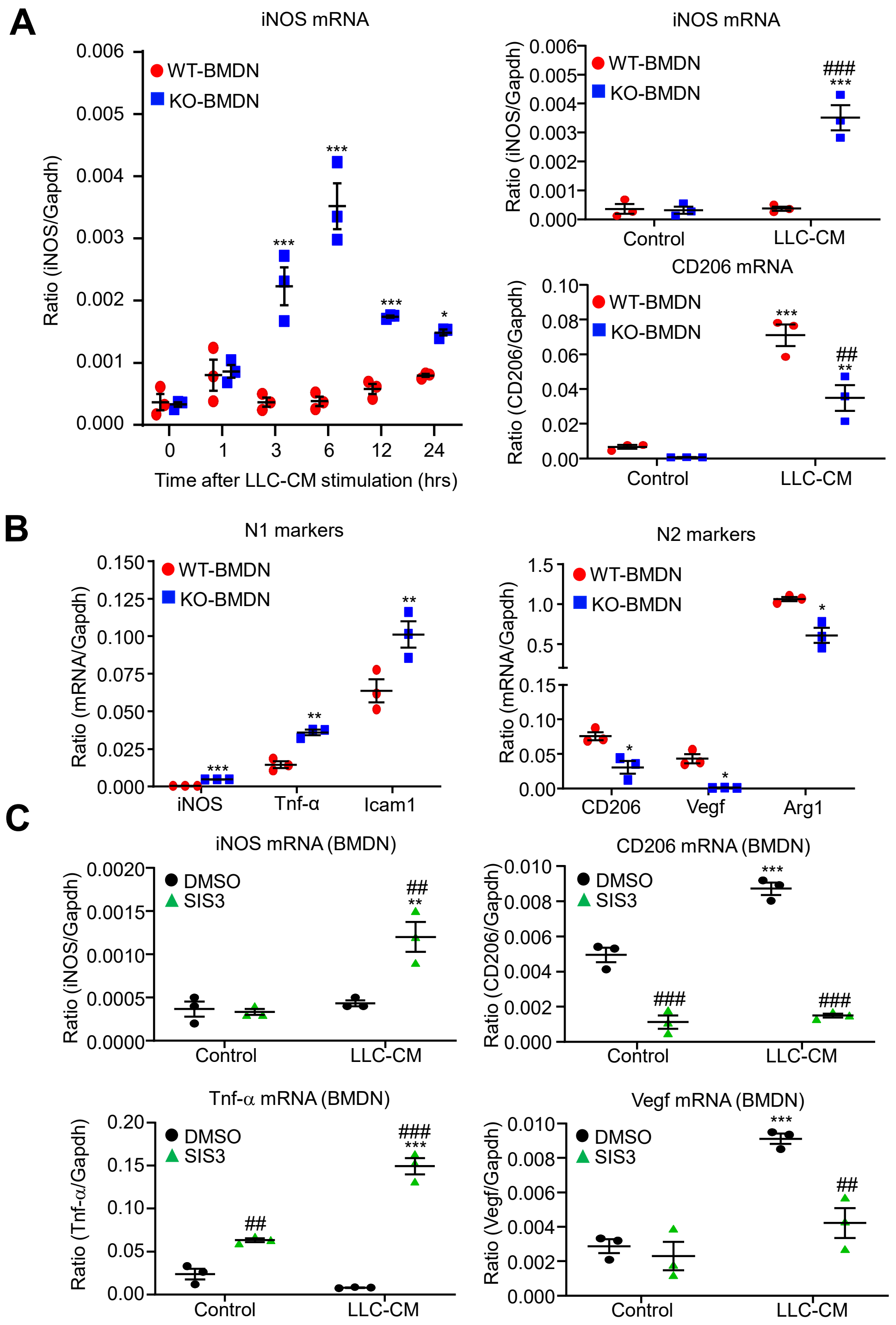

Supplementary Fig 7. Phenotypic changes of wildtype, Smad3-KO BMDNs under cancer condition and LLC-CM stimulated BMDNs under SIS3 treatment *in vitro*. A. iNOS and CD206 expression in BMDN under stimulation with LLC-CM in vitro via PT-PCR. (*p<0.05 ,**p<0.01, ***p<0.001 vs WT-BMDN; ##p<0.01, ###p<0.001 vs Control, n=3 independent samples, one-way ANOVA) **B.** RT-PCR shows an increase in N1 marker (iNOS, Tnf-α, Icam1) mRNA expression in Smad3-KO neutrophils and N2 marker (CD206, Vegf, Arg1) mRNA expression in Smad3-WT neutrophils respectively under 6 hours of LLC-CM stimulation. (*p<0.05 ,**p<0.01, ***p<0.001 vs WT-BMDN, n=3 independent samples, one-way ANOVA). **C**. The pharmaceutical inhibition of Smad3 with Smad3 specific inhibitor SIS3 can enhanced expression of N1 phenotypes (iNOS, Tnf-α) and reduce the expression of N2 phenotype (CD206, Vegf) in the LLC-CM BMDNs in vitro. (***P*<0.01, ****P*<0.001 vs control; ##*P*<0.01, ### *P*<0.001 vs WT-BMDN or 0.05% DMSO, n=3 independent samples, one-way ANOVA). (A-C) Data represents mean ± SEM from three independent experiments. Exact P values of WT-BMDN vs. KO-BMDN are **7A**. *P*=0.0001 (3hrs), *P*=0.0001 (6hrs), *P*=0.0003(12hrs), *P*=0.0453 (24hrs), *P*=0.0001 (KO-BMDN vs. WT-BMDN, LLC-CM, iNOS), *P*=0.0001 (KO-BMDN, Control vs. LLC-CM, iNOS), *P*=0.0036 (KO-BMDN vs. WT-BMDN, LLC-CM, CD206), *P*=0.0052 (KO-BMDN, Control vs. LLC-CM, CD206), *P*=0.0001 (WT-BMDN, Control vs. LLC-CM, CD206), **7B**. *P*=0.0002 (iNOS), *P*=0.0035(Tnf-α), *P*=0.0019 (Icam1), *P*=0.0203 (CD206), *P*=0.0222 (Vegf), *P*= 0.0222(Arg1). Exact P values of SIS3 treatment vs. DMSO are **7C**. *P*=0.0028 (SIS3-BMDN vs. DMSO-BMDN, LLC-CM, iNOS), *P*=0.0013 (SIS3-BMDN, Control vs. LLC-CM, iNOS), *P*=0.0002 (SIS3-BMDN vs. DMSO-BMDN, Control, CD206), *P*=0.0001 (SIS3-BMDN vs. DMSO-BMDN, LLC-CM,CD206), *P*=0.0001 (DMSO-BMDN, Control vs. LLC-CM, CD206), *P*=0.0001 (SIS3-BMDN vs. DMSO-BMDN, LLC-CM, Tnf-α), *P*=0.0057 (SIS3-BMDN vs. DMSO-BMDN, Control, Tnf-α), *P*=0.0001 (SIS3-BMDN, Control vs. LLC-CM, Tnf-α), *P*=0.0031 (SIS3-BMDN vs. DMSO-BMDN, LLC-CM, Vegf), *P*=0.0006 (DMSO-BMDN, Control vs. LLC-CM, Vegf). Source data are provided as a Source Data file.

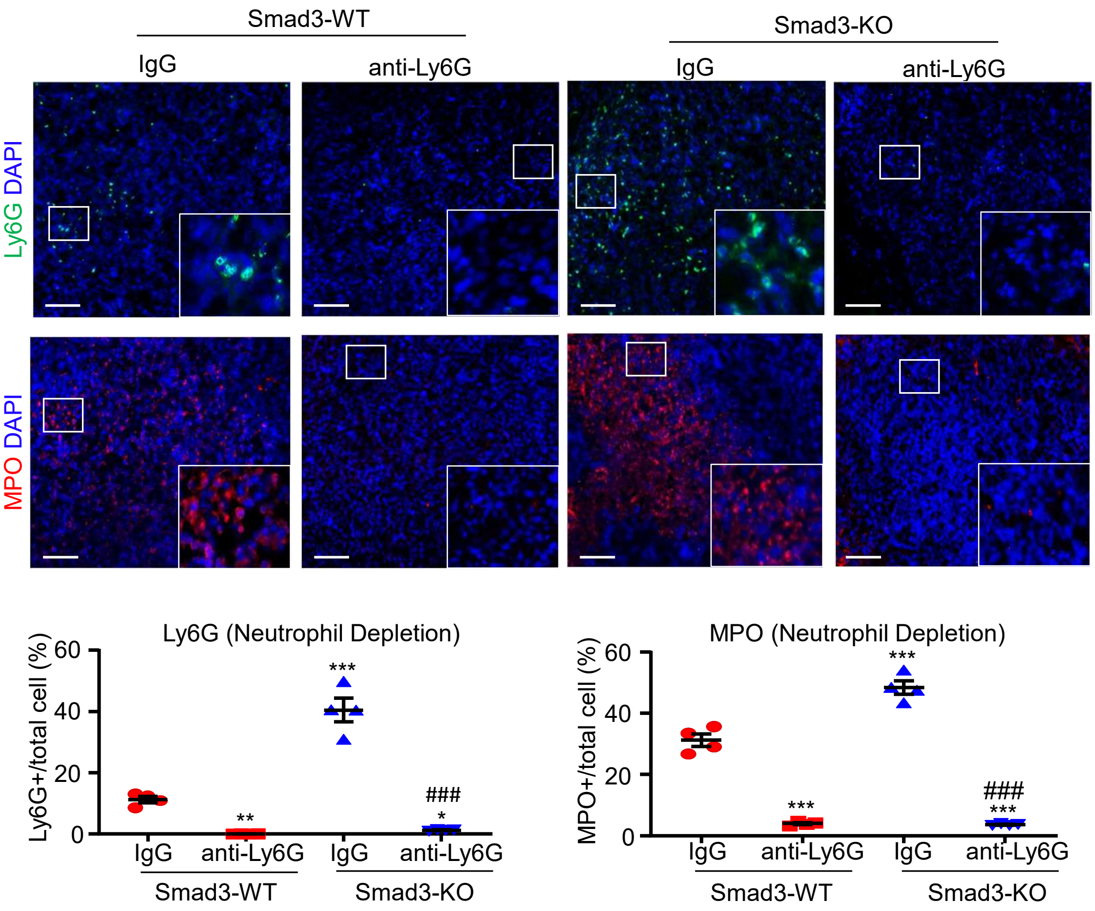

Supplementary Fig 8. Quality controls for the Ly6G-mediated neutrophil depletion of LLC-bearing mice. The anti-Ly6G antibody successfully depleted the neutrophils of LLC-bearing mice, confirming by the reduction of Ly6G^{+} and Myeloperoxidase (MPO) cells with immunofluorescence assay. (*P<0.05, **P <0.01, ***P<0.001 vs IgG-treated Smad3-WT mice; ###P<0.001 vs IgG-treated Smad3-KO mice, one-way ANOVA). Data represents mean ± SEM of 4 mice/group. Scale bar, 50 µm. Exact P values of IgG- vs. anti-Ly6G-treatment are P=0.0001 (IgG KO vs. IgG WT, Ly6G), P=0.0178 (anti-Ly6G KO vs. IgG WT, Ly6G), P=0.0083 (anti-Ly6G WT vs. IgG WT, Ly6G), P=0.0001 (anti-Ly6G KO vs. IgG KO, Ly6G), P=0.0001 (IgG KO vs. IgG WT, MPO), P=0.0001 (anti-Ly6G KO vs. IgG WT, MPO), P=0.0001 (anti-Ly6G WT vs. IgG WT, MPO), P=0.0001 (anti-Ly6G KO vs. IgG KO, MPO). Source data are provided as a Source Data file

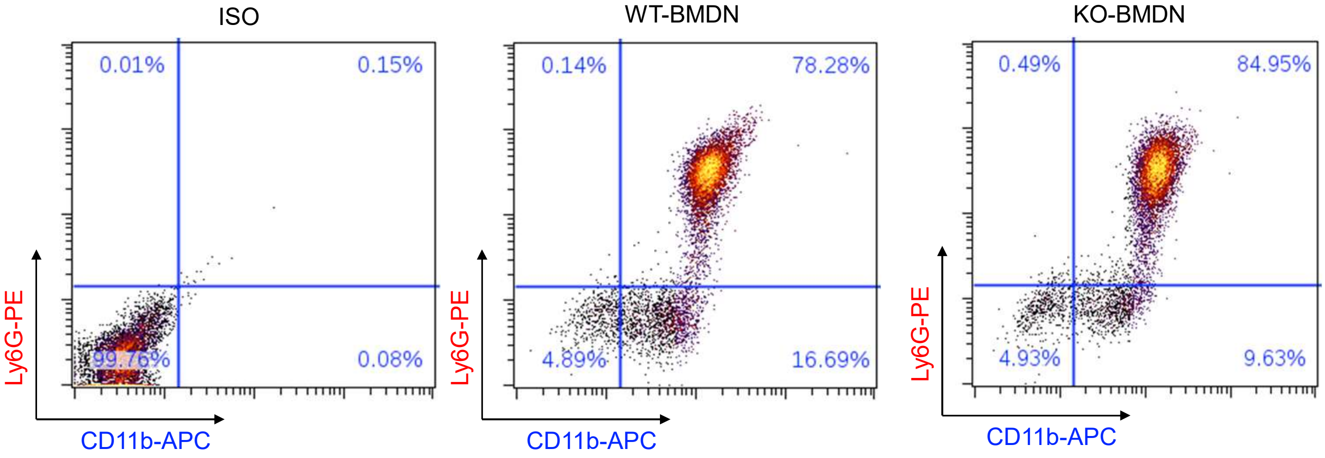

Supplementary Fig 9. Isolation of BM-derived neutrophils *in vitro*.
Flow cytometry shows BM-derived neutrophils from Smad3-WT and -KO mice were successfully isolated by MACS kit.

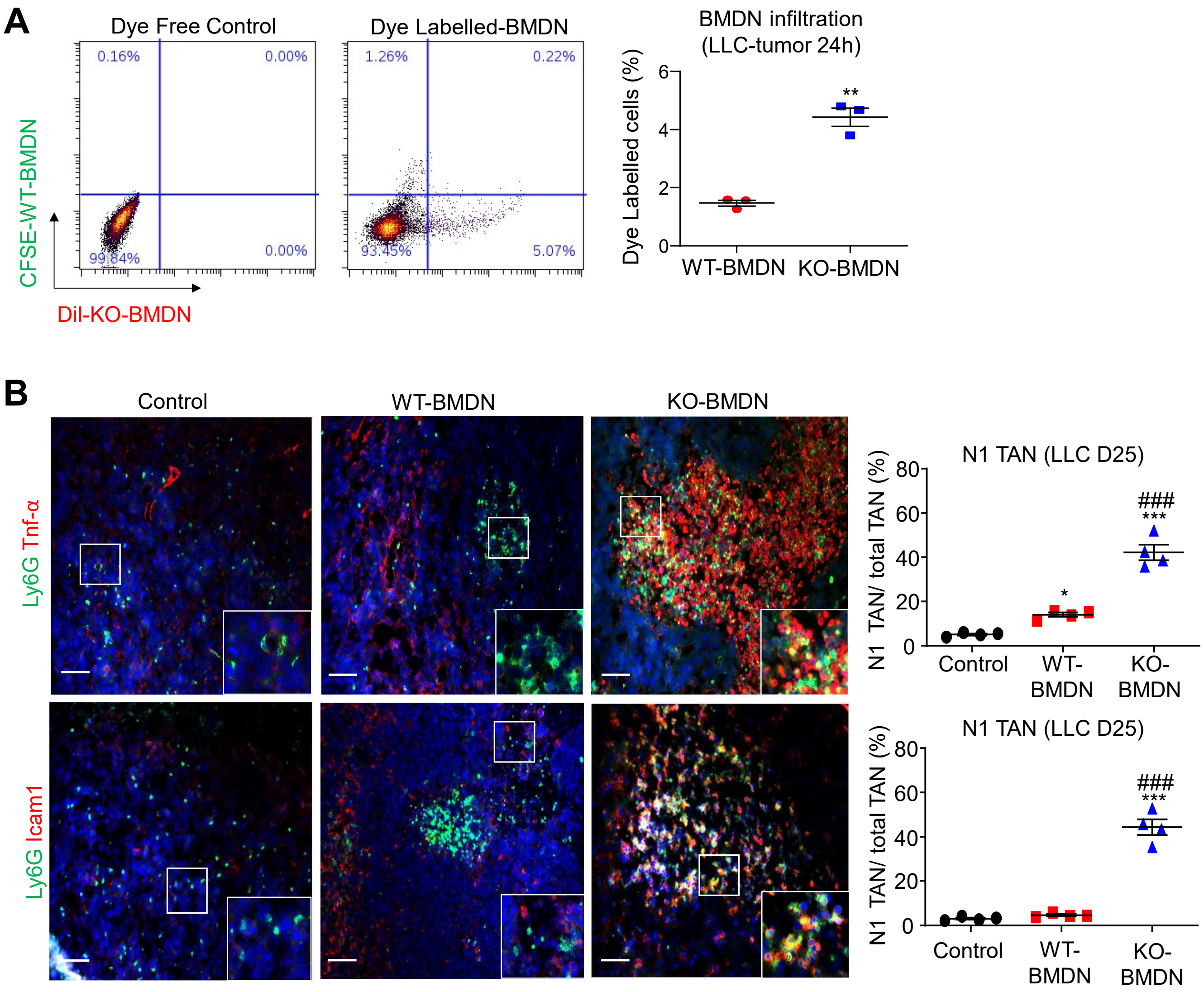

Supplementary Fig 10. Smad3 deficiency enhances tumor infiltration of Smad3-KO BMDNs in the LLC-bearing mice *in vivo*. A. Co-injection of both dye-labelled wildtype and Smad3-KO BMDNs into the same LLC-bearing mice demonstrated the enhanced recruitment of Dil-labelled Smad3-KO BMDNs (Dil-KO-BMDN) compared to the CFSE-labelled wildtype BMDNs (CFSE-WT-BMDN) at 24h after adoptive transfer in the Day 15 LLC-tumor *in vivo*. (**$P<0.01$ vs. WT-BMDN, two tailed t-test). **B.** Adoptive transfer of Smad3-KO BMDNs markedly enhanced the protein expression of additional N1 marker genes Tnf-α and Icam1 in the TME compared to the mice received wildtype BMDNs (WT-BMDN). (*$P<0.05$, ***$P<0.001$ vs. Control, ### $P<0.001$ vs. WT-BMDN, one-way ANOVA). Scale bars, 50 µm. (A) Data represents mean ± SEM of 3 mice/group. (B) Data represents mean ± SEM of 4 mice/group. Exact P value of WT-BMDN vs. KO-BMDN infiltration is **10A**. P=0.0029. Exact P values of BMDN adoptive transfer are **10B**. P=0.0001 (KO-BMDN vs. WT-BMDN, Tnf-α+ TAN), P=0.0001 (KO-BMDN vs. Control, Tnf-α+ TAN), P=0.0397 (WT-BMDN vs. Control, Tnf-α+ TAN), P=0.0001 (KO-BMDN vs. WT-BMDN, Icam1+ TAN), P=0.0001 (KO-BMDN vs. Control, Icam1+ TAN). Source data are provided as a Source Data file

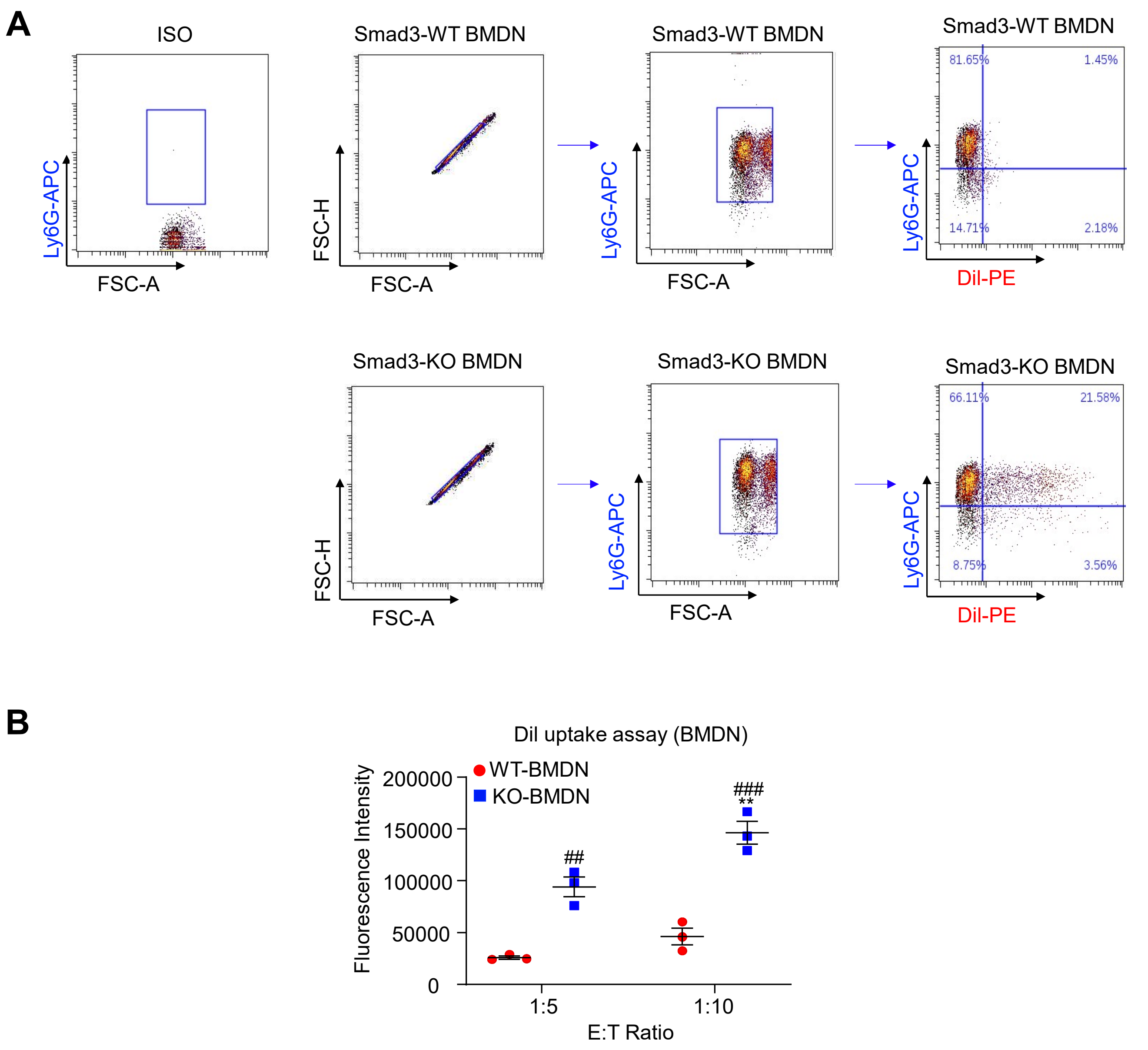

Supplementary Fig 11. Smad3 deficiency enhances phagocytosis of Smad3-KO BMDNs towards LLC *in vitro*. Flow cytometry **(A)** and Dil uptake assay **(B)** shows 6 hours co-culture of wildtype and Smad3-KO BMDNs with dye-labelled LLC cell respectively demonstrated the enhanced phagocytosis activity of Smad3-KO BMDNs compared to the wildtype BMDNs *in vitro*. (***P*<0.01 vs 1:5 group, ##*P*<0.01, ###*P*<0.001 vs Smad3-WT BMDN, n=3 independent samples, one-way ANOVA) . (B) Data represents mean ± SEM from three independent experiments. Exact P value of WT-BMDN vs. KO-BMDN dil uptake assay is **11B**. *P*=0.0019 (1:5), 2B. *P*=0.0001 (1:10), *P*=0.0096(KO-BMDN, 1:5 vs. 1:10 group). Source data are provided as a Source Data file

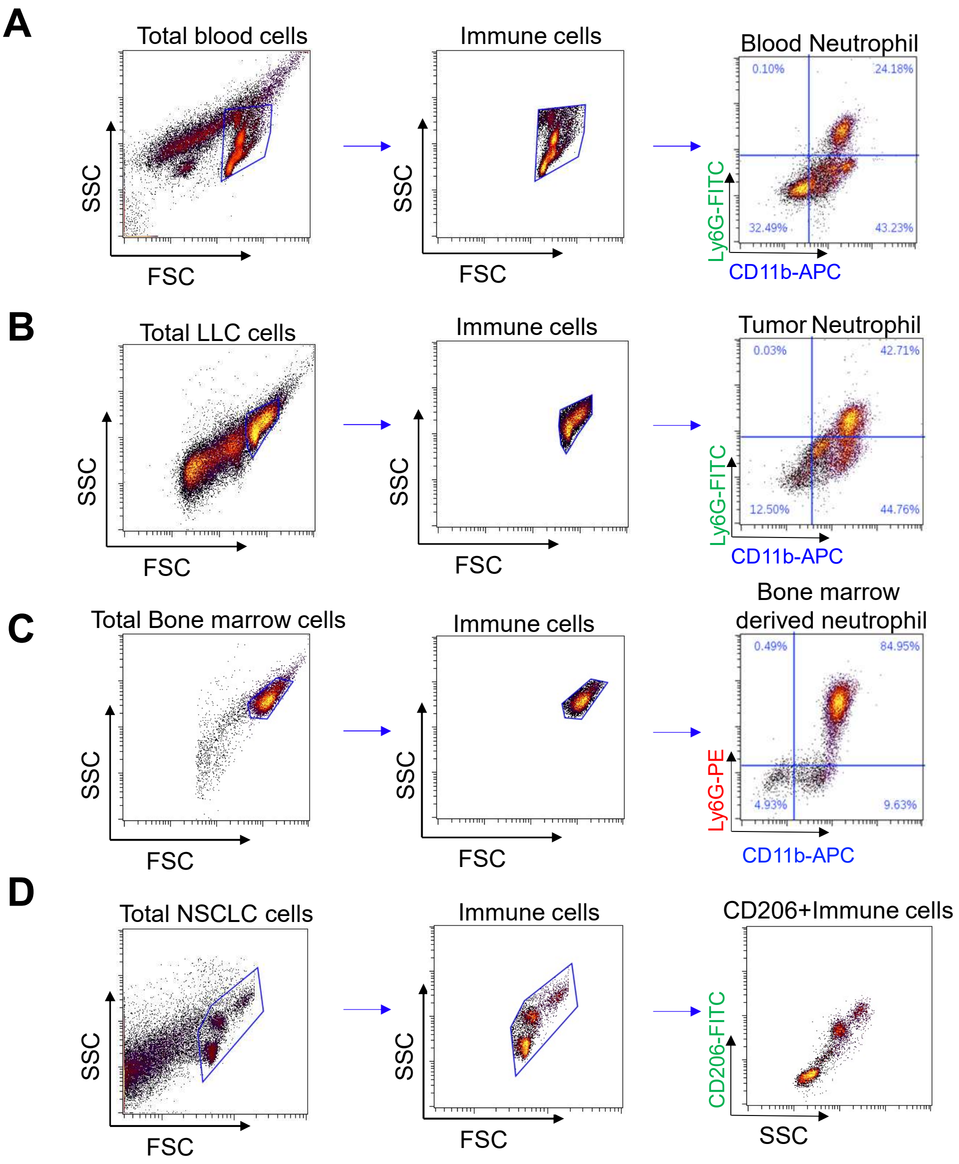

Supplementary Fig 12. Representative flow cytometry gating.

Flow cytometric gating hierarchy used for **(A)** mice blood immune cells applied in Figure. 2B, **(B)** mice LLC tumor immune cells applied in Figure 2B, 2E and Supplementary Fig 10A, **(C)** mice bone marrow cell applied in Supplementary Fig 9, 11A and **(D)** human NSCLC immune cell applied in Supplementary Fig 1A.

**LUAD Tissue Array**	**Clinical information**
Tumor Stage (1-4)	1(37)<br>2-4(35)
Tumor size (cm)	≤3.5(43)<br>>3.5(29)
N1 TAN/Total TAN (%)	≥10 (42)<br><10(30)

Supplementary Table 1. Clinical information and demographic data of LUAD cohort.

Quality statistics of scRNA-seq datasets		
	**Smad3-WT TAN**	**Smad3-KO TAN**
**Source**	FACS of DAPI^{-} CD11b^{+} Ly6G^{+} cells from LLC tumors on Smad3-WT/KO mice (pooled from 8 tumors)	
**Total cells deteceted**	4116	2,514
**Total reads**	549,316,839	540,958,267
**Total Genes**	17,872	16,523
**Mean Reads per Cell**	133,458	215,178
**Median Genes per Cell**	781	554
**Median UMI Counts per Cell**	1,671	1,187
**Valid barcodes**	95.8%	95.2%

Supplementary Table 2. Quality statistics of TAN-specific10X scRNA-seq datasets.

Gene	Cluster P1	Cluster P2	Cluster P3	Cluster P4	Cluster P5	Cluster P6	Cluster P7	Cluster P8
**1**	Igfbp4	Plac8	H2-Eb1	Mmp9	Camp	Cxcl3	Ifit3b	Il1a
**2**	Rps7	Ly6c2	H2-Ab1	Slfn1	Ngp	Hcar2	Ifit3	Tnfaip3
**3**	Npm1	Ifi27l2a	H2-Aa	Csf1	Ltf	Gm5483	Ifit1	Lcn2
**4**	Rps2	Irf7	C1qa	Egr1	Pglyrp1	Ccl3	Cmpk2	Gm3839
**5**	Rpl13	Arg1	C1qb	Txnip	Retnlg	Ccl4	Gm13822	Gadd45b
**6**	Rpl12	Hmox1	C1qc	Il1r2	Wfdc21	Slc7a11	Cxcl10	Ccrl2
**7**	Eef1g	Thbs1	Cd74	Cd24a	S100a8	Hilpda	Oasl1	Tnf
**8**	Serbp1	Ccl9	Ccl8	Btg2	S100a9	Egr1	Rsad2	Slpi
**9**	Hspe1	Lgals3	Apoe	Clec4n	Ifitm6	Il1rn	Isg20	Cd274
**10**	Rpl36a	Ctsc	Lyz2	Sell	G0s2	Ier3	Isg15	Acod1

Supplementary Table 3. Top 10 up-regulated genes in each TAN cluster of the scRNA-seq dataset .

Gene Name	Forward (5' to 3')	Reverse (5' to 3')
Gapdh (Mouse)	GCATGGCCTTCCGTGTTC	GATGTCATCATACTTGGCAGGTTT
iNOS (Mouse)	ACATGCAGAATGAGTACCGG	TCAACATCTCCTGGTGGAAC
TNF-α (Mouse)	CATCTTCTCAAAATTCGAGTGACAA	TGGGAGTAGACAAGGTACAACCC
Icam1(Mouse)	CATGCCTTAGCAGCTGAACA	AGCTTGCACGACCCTTCTAA
Cd206 (Mouse)	GTCAGAACAGACTGCGTGGA	AGGGATCGCCTGTTTTCCAG
Vegf (Mouse)	ACCGTTGACAGAACAGTCCTTAATC	GACCCAAAGTGCTCCTCGAA
Arg1 (Mouse)	AGCTCTGGGAATCTGCATGG	ATGTACACGATGTCTTTGGCAGATA
GAPDH (human)	TGAAGGTCGGAGTCAACGGATTTGGT	CATGTGGGCCATGAGGTCCACCAC
SMAD3 (human)	TGAGGCTGTCTACCAGTTGACC	GTGAGGACCTTGTCAAGCCACT

Supplementary Table 4. Real-time PCR primer sequences of the mouse and human mRNAs.
