# Supplementary material for: Smad3 is essential for polarization of tumor-associated neutrophils in non-small cell lung carcinoma
Source: Nat Commun. 2023 Mar 31;14:1794. doi: 10.1038/s41467-023-37515-8 (PMC10066366; doi:10.1038/s41467-023-37515-8)
Supplement: Supplementary file 2 — Description of Additional Supplementary Files [file 41467_2023_37515_MOESM2_ESM.pdf]

Description of Additional Supplementary Files

File Name: Supplementary Data 1

Description: Clinical information and the relative marker quantification of Opal tissue array

File Name: Supplementary Data 2

Description: Single cell RNA-seq related gene list and expression level for Figure 3A, 3C, 3D, 3F,3G and 4E

File Name: Supplementary Data 3

Description: ChIP-seq related gene list
