# Supplementary material for: Smad3 is essential for polarization of tumor-associated neutrophils in non-small cell lung carcinoma
Source: Nat Commun. 2023 Mar 31;14:1794. doi: 10.1038/s41467-023-37515-8 (PMC10066366; doi:10.1038/s41467-023-37515-8)
Supplement: Supplementary file 6 — Reporting Summary [file 41467_2023_37515_MOESM6_ESM.pdf]

nature portfolio

Corresponding author(s): Dr Patrick Ming-Kuen Tang

Last updated by author(s): Mar 7, 2023

Reporting Summary

Nature Portfolio wishes to improve the reproducibility of the work that we publish. This form provides structure for consistency and transparency in reporting. For further information on Nature Portfolio policies, see our Editorial Policies and the Editorial Policy Checklist.

Statistics

For all statistical analyses, confirm that the following items are present in the figure legend, table legend, main text, or Methods section.

n/a	Confirmed	
☐	☒	The exact sample size (*n*) for each experimental group/condition, given as a discrete number and unit of measurement
☐	☒	A statement on whether measurements were taken from distinct samples or whether the same sample was measured repeatedly
☐	☒	The statistical test(s) used AND whether they are one- or two-sided<br>*Only common tests should be described solely by name; describe more complex techniques in the Methods section.*
☒	☐	A description of all covariates tested
☐	☒	A description of any assumptions or corrections, such as tests of normality and adjustment for multiple comparisons
☐	☒	A full description of the statistical parameters including central tendency (e.g. means) or other basic estimates (e.g. regression coefficient) AND variation (e.g. standard deviation) or associated estimates of uncertainty (e.g. confidence intervals)
☐	☒	For null hypothesis testing, the test statistic (e.g. *F*, *t*, *r*) with confidence intervals, effect sizes, degrees of freedom and *P* value noted<br>*Give P values as exact values whenever suitable.*
☒	☐	For Bayesian analysis, information on the choice of priors and Markov chain Monte Carlo settings
☒	☐	For hierarchical and complex designs, identification of the appropriate level for tests and full reporting of outcomes
☒	☐	Estimates of effect sizes (e.g. Cohen's *d*, Pearson's *r*), indicating how they were calculated

Our web collection on statistics for biologists contains articles on many of the points above.

Software and code

Policy information about availability of computer code

Data collection	-Zeiss Axio Observer.Z1<br>-Zeiss LSM 880 laser scanning confocal microscope<br>-Mantra quantitative pathology workstation (Akoya biosciences)<br>-LSRFortessa (Becton Dickinson)<br>-illumina HiSeq<br>-illumina NovaSeq 6000
Data analysis	-Prism program (Prism 5.0,9.0 GraphPad Software, San Diego, CA)<br>-ZEN lite image analysis software 2.4<br>-inForm image analysis software (Akoya biosciences) 2.6<br>-EaSeq 1.111<br>-Genomic Regions Enrichment of Annotations Tool (GREAT) 4.0.4<br>-Trawler (trawler.erc.monash.edu.au) 2.0<br>-R studio (v1.3.1073)<br>-Loupe Cell Browser 6.0<br>-Cytobank platform (cytobank.org) 9.4

For manuscripts utilizing custom algorithms or software that are central to the research but not yet described in published literature, software must be made available to editors and reviewers. We strongly encourage code deposition in a community repository (e.g. GitHub). See the Nature Portfolio guidelines for submitting code & software for further information.

Data

Policy information about availability of data

All manuscripts must include a data availability statement. This statement should provide the following information, where applicable:

- Accession codes, unique identifiers, or web links for publicly available datasets
- A description of any restrictions on data availability
- For clinical datasets or third party data, please ensure that the statement adheres to our policy

The raw single cell RNA-seq and ChIP-seq datasets are available in the Gene Expression Omnibus and ArrayExpress database under the accession number GSE186530 and E-MTAB-12740 respectively. The human NSCLC publicly available data used in this study are available in the 10X Geomics database[https://www.10xgenomics.com/resources/datasets/40-k-mixture-of-nsclc-dt-cs-from-7-donors-3-ht-v-3-1-3-1-high-6-1-0]. Source data are provided in this paper.

Human research participants

Policy information about studies involving human research participants and Sex and Gender in Research.

Reporting on sex and gender	Patient sex information was not considered in study design.
Population characteristics	Clinical information are listed in Supplementary Table 1 and Supplementary Data 1
Recruitment	Participants were recruited by face to face. Written informed consent were obtained from all participants after they notice the study detail and agree to involve. To avoid self-selection bias, the researcher who analyses the data was not involved in the recruitment and consent process.
Ethics oversight	The study was conducted according to the principles of the Declaration of Helsinki.This study was approved by the Joint Chinese University of Hong Kong – New Territories East Cluster Clinical Research Ethics Committee (CREC Ref. No.: 2018.054).

Note that full information on the approval of the study protocol must also be provided in the manuscript.

Field-specific reporting

Please select the one below that is the best fit for your research. If you are not sure, read the appropriate sections before making your selection.

[x] Life sciences [ ] Behavioural & social sciences [ ] Ecological, evolutionary & environmental sciences

For a reference copy of the document with all sections, see nature.com/documents/nr-reporting-summary-flat.pdf

Life sciences study design

All studies must disclose on these points even when the disclosure is negative.

Sample size	Sample size was determined on calculations based on our previous published data (https://www.nature.com/articles/ncomms14677) and power calculator from Animal Experimentation Ethics Committee(CUHK), showing that a sample size of n = 3 to 5 mice per group are sufficient to attain statistical significance of p<0.05. Triplicate experiments were applied in in-vitro experiments. For Single cell RNA-seq experiments, 8 tumors per genotype were pooled. For ChIP-seq experiments, each group of bone marrow derived neutrophils was pooled from at least three mice.<br><br>A total of 72 lung adenocarcinoma specimens from patients with complete patient demographic data (stage, mutation) were retrieved from LUAD cohort, Prince of Wales Hospital, Hong Kong.
Data exclusions	No data were excluded
Replication	For all data presented in the manuscript, we examined at least three independent biological samples (three different mice at one time) to ensure the reproducibility. For each series of the experiments, all attempts at replication were successful.
Randomization	Animals were randomly assigned to the experimental groups. Smad3-KO and Smad3-WT mice were determined by genotype.
Blinding	Investigators were not blinded to allocation of mice for experimental groups because this choice was driven by genotype (Smad3-KO and Smad3-WT). Experiments that were consistently blinded included Immunohistochemistry, Immunofluorescence, FACS and qPCR.

Reporting for specific materials, systems and methods

We require information from authors about some types of materials, experimental systems and methods used in many studies. Here, indicate whether each material, system or method listed is relevant to your study. If you are not sure if a list item applies to your research, read the appropriate section before selecting a response.

Materials & experimental systems

n/a	Involved in the study
☐	☒ Antibodies
☐	☒ Eukaryotic cell lines
☒	☐ Palaeontology and archaeology
☐	☒ Animals and other organisms
☒	☐ Clinical data
☒	☐ Dual use research of concern

Methods

n/a	Involved in the study
☐	☒ ChIP-seq
☐	☒ Flow cytometry
☒	☐ MRI-based neuroimaging

Antibodies

Antibodies used

mCD206 (1:100, 141706, BioLegend, clone:C068C2), hCD206 (1:100, sc-376232, Santa Cruz,clone:C-10),hCD206-FITC(1:100, 321104, BioLegend, clone:15C2), iNOS (1:100, sc-7271, Santa Cruz,clone:C-11), TNF-α (1:100, sc-52746, Santa Cruz,clone:C-4), Icam1 (1:100, sc-8439, Santa Cruz,clone:G-5), p-Smad3 (paraffin section, 1:100, sc-517575, Santa Cruz,clone:1D9), CD16b-PE (1:100, 550868, BD Biosciences,clone: CLBgran11.5), Ly6G-FITC (1:100, 127606, BioLegend,clone:1A8), Ly6G-PE (1:100, 127608, BioLegend,clone:1A8), Ly6G-APC (1:100, 127614, BioLegend,clone:1A8), p-Smad3 (1:200, 600-401-919, Rockland,Polyclonal), CD11b-APC (1:100, 553311, BD Biosciences,clone:M1/70 (RUO)). CD68-APC(1:100, 333810, BioLegend, clone:Y1/82A). Anti-Smad3 (1:100, 9523S, Cell Signaling technology,clone:C67H9) , IgG Isotype Control （3900S, Cell signaling technology,clone:DA1E), EnVision+system-HRp-Labelled Polymer Anti-mouse(100 μL per section, K4003, Dako), EnVision+system-HRp-Labelled Polymer Anti-Rabbit(100 μL per section, K4003, Dako), Goat anti-Rat IgG (H+L) Secondary Antibody, HRP(1:200,31470, Thermo fisher).Goat anti-Mouse IgG (H+L) Cross-Adsorbed Secondary Antibody, Alexa Fluor™ 488(A-11001, Invitrogen), Goat anti-Mouse IgG (H+L) Highly Cross-Adsorbed Secondary Antibody, Alexa Fluor™ 546(A-11035, Invitrogen), Goat anti-Rabbit IgG (H+L) Highly Cross-Adsorbed Secondary Antibody, Alexa Fluor™ 546(A-11035, Invitrogen), Goat anti-Rabbit IgG (H+L) Highly Cross-Adsorbed Secondary Antibody, Alexa Fluor™ Plus 647 (A32733, Invitrogen), Goat anti-Mouse IgG (H+L) Highly Cross-Adsorbed Secondary Antibody, Alexa Fluor™ Plus 647(A32728, Invitrogen)

Validation

All antibodies used in our study are commercially purchased from credible sources and have been validated by the manufacturer and other investigator. All antibody validation can be found on the manufacturer website.
mCD206-PE validated on mouse macrophages (https://www.biolegend.com/en-us/products/pe-anti-mouse-cd206-mmr-antibody-7424?GroupID=BLG9506)
hCD206 validated on human lung tissue (https://www.scbt.com/p/cd206-antibody-c-10)
hCD206-FITC validated on human peripheral blood (https://www.biolegend.com/en-us/search-results/fitc-anti-human-cd206-mmr-antibody-2993)
iNOS validated on RAW 264.7 cell and human heart muscle tissue (https://www.scbt.com/p/nos2-antibody-c-11)
TNF-α validated on mouse heart tissue (https://www.scbt.com/p/tnfalpha-antibody-52b83)
Icam1 validated on mouse colon tissue(https://www.scbt.com/p/icam-1-antibody-g-50)
p-Smad3 (Santa Cruz) validated on human Lung cancer and mouse hepatocellular carcinoma (https://www.scbt.com/p/p-smad3-antibody-1d9)
CD16b-PE validated on peripheral blood granulocytes (https://www.bdbiosciences.com/en-eu/products/reagents/flow-cytometry-reagents/research-reagents/single-color-antibodies-ruo/pe-mouse-anti-human-cd16b.550868)
Ly6G-FITC validated on mouse bone marrow cells (https://www.biolegend.com/en-us/products/fitc-anti-mouse-ly-6g-antibody-4775?GroupID=BLG5803)
Ly6G-PE validated on mouse bone marrow cells (https://www.biolegend.com/en-us/products/pe-anti-mouse-ly-6g-antibody-4777?GroupID=BLG5803)
Ly6G-APC validated on mouse bone marrow cells (https://www.biolegend.com/fr-lu/products/apc-anti-mouse-ly-6g-antibody-6115)
CD11b-APC validated on bone-marrow myeloid cells (https://www.bdbiosciences.com/en-au/products/reagents/flow-cytometry-reagents/research-reagents/single-color-antibodies-ruo/pe-rat-anti-cd11b.553311)
p-Smad3 (Rockland) validated on human brain and cancer (https://www.rockland.com/categories/primary-antibodies/smad3-phospho-s423-phospho-s425-antibody-600-401-919/)
CD68-APC validated on human peripheral blood monocytes (https://www.biolegend.com/en-us/products/apc-anti-human-cd68-antibody-6542?GroupID=BLG10058)

Eukaryotic cell lines

Policy information about cell lines and Sex and Gender in Research

Cell line source(s)

Lewis lung carcinoma LLC (CRL-1642, ATCC)

Authentication	None of the cell line used were authenticated
Mycoplasma contamination	The cell line were not tested for Mycoplasma contamination. Mycoplasma contamination was excluded through PCR-based and luminescence-based mycoplasma assays by the manufacturer
Commonly misidentified lines (See ICLAC register)	Lewis lung carcinoma LLC (CRL-1642, ATCC) is not list in version 12 of ICLAC Register of Misidentified Cell Lines

Animals and other research organisms

Policy information about studies involving animals; ARRIVE guidelines recommended for reporting animal research, and Sex and Gender in Research

Laboratory animals	Smad3-wildtype (Smad3+/+), Smad3-deficient (Smad3-/-) mice on C57BL/6J background (both sexes, aged 8-12 weeks) and NOD.Cg-PrkdcscidIl2rgtm1Wjl/SzJ mice (NSG mice, male, aged 8-10-weeks) were used in this study. All mice were provided by the Laboratory Animal Service Center of CUHK and maintained at 22–23 °C, <70% relative humidity with an alternating 12 h light/dark cycle with free access to standard diet and water.
Wild animals	No wild animal involve
Reporting on sex	both sex involve in the animal study
Field-collected samples	The study did not involve field-collected samples
Ethics oversight	All experimental procedures were approved by the Animal Experimentation Ethics Committee of the Chinese University of Hong Kong (CUHK) and were carried out in accordance with the Guide for the Care and Use of Laboratory Animals. (AEEC Ref No.: 18/005/GRF).

Note that full information on the approval of the study protocol must also be provided in the manuscript.

ChIP-seq

Data deposition

- [x] Confirm that both raw and final processed data have been deposited in a public database such as GEO.
- [x] Confirm that you have deposited or provided access to graph files (e.g. BED files) for the called peaks.

Data access links<br>*May remain private before publication.*	The raw ChIP-seq datasets are available in the ArrayExpress database under the accession number E-MTAB-12740.
Files in database submission	Smad3_ChIP_CM_1.fq.gz<br>Smad3_ChIP_CM_2.fq.gz<br>Smad3_ChIP_Co_1.fq.gz<br>Smad3_ChIP_Co_2.fq.gz<br>Smad3_Input_CM_1.fq.gz<br>Smad3_Input_CM_2.fq.gz<br>Smad3_Input_Co_1.fq.gz<br>Smad3_Input_Co_2.fq.gz<br><br>Smad3_ChIP_CM.bed<br>Smad3_ChIP_Co.bed
Genome browser session<br>(e.g. UCSC)	Genomic Regions Enrichment of Annotations Tool (GREAT)<br>http://great.stanford.edu/public/html/

Methodology

Replicates	Input and Smad3-IP is pooled from 4 individual sample
Sequencing depth	6GB
Antibodies	Anti-Smad3 (1:100, 9523S, Cell signaling technology), IgG Isotype Control (1:1000, 3900S, Cell signaling technology)
Peak calling parameters	Mapped against GRCm38 (mm10) mus musculus genome using bowtie2. Peak called using model-based analysis for ChIP-Seq (MACS) with default parameters
Data quality	Peaks that were detected in the input sample were filtered out.

Software	Binding regions were visualized by EaSeq, and annotated by Genomic Regions Enrichment of Annotations Tool (GREAT) for DAVID-GO enrichment analysis. Motif analysis was performed in Trawler (trawler.erc.monash.edu.au) with default parameters.

Flow Cytometry

Plots

Confirm that:

- [x] The axis labels state the marker and fluorochrome used (e.g. CD4-FITC).
- [x] The axis scales are clearly visible. Include numbers along axes only for bottom left plot of group (a 'group' is an analysis of identical markers).
- [x] All plots are contour plots with outliers or pseudocolor plots.
- [x] A numerical value for number of cells or percentage (with statistics) is provided.

Methodology

Sample preparation	Tissues isolated from tumor-bearing mice were mechanistically dissociated, digested by Liberase™ TM (Roche), filtered by 40-µm nylon mesh, and fixed with IC Fixation Buffer (eBioscience) according to manufacturer's protocol to prepare single-cell suspension.
Instrument	LSRFortessa (Becton Dickinson)
Software	Flow cytometric data were acquired on LSRFortessa (Becton Dickinson) and analyzed in the Cytobank platform (cytobank.org) for quantitative analysis
Cell population abundance	Profiling of neutrophil was performed on single cell suspensions created from LLC tumors grown in mice. After FSC/SSC gating 10,000 cells was collected for experiments.
Gating strategy	Use FSC/SSC to gate the stating population, use ISO control without antibody staining as negative control. Gating strategy is shown in Supplementary Fig 12.

- [x] Tick this box to confirm that a figure exemplifying the gating strategy is provided in the Supplementary Information.
